# The unique fibrilar to platy nano- and microstructure of twinned rotaliid foraminiferal shell calcite

**DOI:** 10.1038/s41598-022-25082-9

**Published:** 2023-02-07

**Authors:** J. Lastam, E. Griesshaber, X. Yin, U. Rupp, I. Sánchez-Almazo, M. Heß, P. Walther, A. Checa, W. W. Schmahl

**Affiliations:** 1 Forschungszentrum Jülich, Institut für Energie und Klimaforschung, IEK-2, 52425 Jülich, Germany; 2 Department für Geo- und Umweltwissenschaften, Ludwig-Maximilians-Universität München, 80333 Munich, Germany; 3 Zentrale Einrichtung Elektronenmikroskopie, Universität Ulm, 89081 Ulm, Germany; 4 Centro de Instrumentación Científica, Universidad de Granada, 18071 Granada, Spain; 5 Biozentrum LMU München, 82152 Planegg-Martinsried, Germany; 6 Departamento de Estratigrafía y Paleontología, Universidad de Granada, 18071 Granada, Spain; 7 Instituto Andaluz de Ciencias de la Tierra, CSIC-Universidad de Granada, 18100 Armilla, Spain

**Keywords:** Biological techniques, Structural biology, Systems biology, Biogeochemistry, Climate sciences, Environmental sciences, Ocean sciences, Materials science, Nanoscience and technology

## Abstract

Diversification of biocrystal arrangements, incorporation of biopolymers at many scale levels and hierarchical architectures are keys for biomaterial optimization. The planktonic rotaliid foraminifer *Pulleniatina obliquiloculata* displays in its shell a new kind of mesocrystal architecture. Shell formation starts with crystallization of a rhizopodial network, the primary organic sheet (POS). On one side of the POS, crystals consist of blocky domains of 1 μm. On the other side of the POS crystals have dendritic-fractal morphologies, interdigitate and reach sizes of tens of micrometers. The dendritic-fractal crystals are twinned. At the site of nucleation, twinned crystals consist of minute fibrils. With distance away from the nucleation-site, fibrils evolve to bundles of crystallographically well co-oriented nanofibrils and to, twinned, platy-blade-shaped crystals that seam outer shell surfaces. The morphological nanofibril axis is the crystallographic c-axis, both are perpendicular to shell vault. The nanofibrillar calcite is polysynthetically twinned according to the 60°/[100] (= m/{001}) twin law. We demonstrate for the twinned, fractal-dendritic, crystals formation at high supersaturation and growth through crystal competition. We show also that c-axis-alignment is already induced by biopolymers of the POS and is not simply a consequence of growth competition. We discuss determinants that lead to rotaliid calcite formation.

## Introduction

Foraminifera are single-celled organisms that live in a wide range of marine environments^1–5^. The majority of foraminifera protect their cytoplasm with a chambered shell mineralized with calcium carbonate (e.g.^6–10^). Foraminifera shells exhibit a large variety of chamber and shell morphologies^3,11–14^. The diversity in shell form, their widespread presence in almost all marine environments, their well-documented fossil record renders this group of protozoa as one of the best-suited taxa for assessments of global carbonate budgets and evolutionary, stratigraphic, paleoecologic studies and paleoclimate reconstructions^3,9,12,15–17^.

Calcite crystal organization was investigated for the shell of the benthic rotaliid species *Amphistegina lessonii* (d’Orbigny 1826), *Amphistegina lobifera* (Larsen 1976) and *Ammonia tepida* (Cushman 1926)^18^. It was shown^18^ that the crystals are mesocrystals and that *A. lessonii*, *A. lobifera,* and *A. tepida* form their shell of crystals having one of the two morphologies: (1) blocky, few micrometer-sized crystallites, and (2) larger, tens of micrometers-sized, dendritic-fractal crystals with an internal nanofibrillar/nanoplaty structure. These two different types of nano- and microstructures are delimited from each other through the primary organic sheet (POS), the biopolymer network where nucleation takes place and shell mineralization starts.

Here we investigate the nanometer-scale structure and sub-micrometer-scale crystallographic organization of planktonic rotaliid foraminiferal shell calcite. We choose for our study the planktonic species *Pulleniatina obliquiloculata*, family Pulleniatinidae, order Rotaliida (Parker and Jones, 1865). *P. obliquiloculata* lives in environments of up to 100 m water depth and is one of the most abundant planktonic species of tropical oceans^19,20^. The shells of *P. obliquiloculata* are widely used for studies investigating environmental controls on shell calcification and for assessments of planktonic foraminiferal shell contribution to the global terrestrial carbon cycle^19,20^. We discuss crystal nucleation and growth of *P. obliquiloculata* calcite and show that within the biopolymer network that templates nucleation both main crystal morphologies that comprise the shell become developed. As most of the shell is formed of dendritic-fractal crystals, we detail these in particular and demonstrate that the latter crystals nucleate at high supersaturation and pH and grow to larger crystals through a competitive growth process. The latter process contributes significantly to the formation of the texture pattern (cylindrical texture) of the crystal assembly. We show that the dendritic-fractal crystals have a specific and unique nano- and microstructure and demonstrate that these crystals consist of twinned calcite. Based on our microstructural-crystallographic observations we discuss the main determinants that initiate, control and guide *P. obliquiloculata* shell calcite formation.

## Results

**Figure 1:**
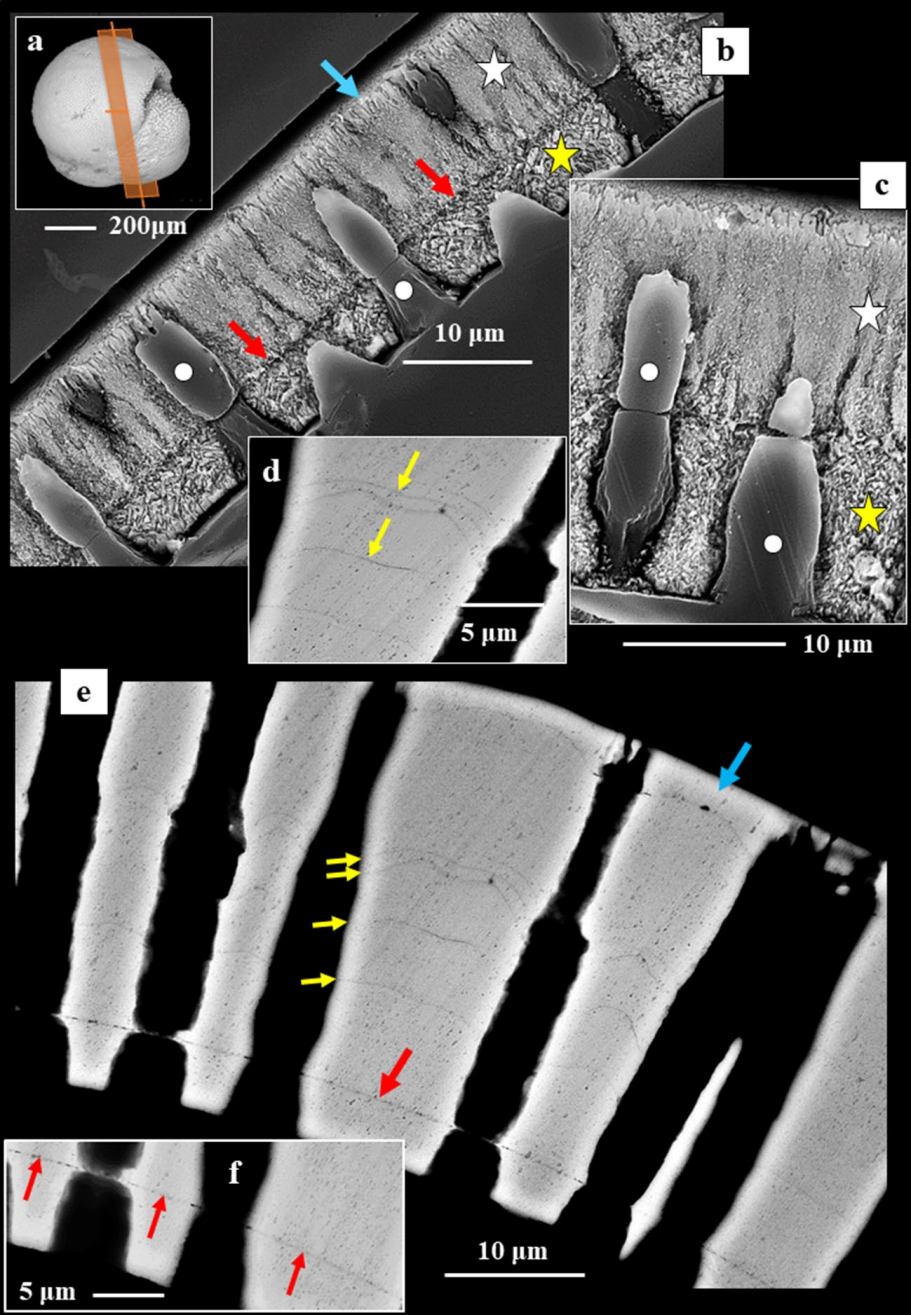
BSE images of shell cross-sections depicting the internal structure of *Pulleniatina obliquiloculata* shells. (**a**) mode of sectioning the shell. (**b**, **c**) BSE images of etched shell surfaces. (**d**, **e**, **f**) BSE images of highly polished, not etched, surfaces; these surfaces were used for EBSD measurements. Well visible is the subdivision of the shell wall by the primary organic sheet (POS) (red arrows in (**b**, **e**, **f**)) into two layers (**b**, **c**). These have different microstructures (white and yellow stars in (**b**) and (**c**)). White dots in (**b**, **c**) indicate the pores that are filled with EPON resin. The course of the POS is very evenly (e.g. (**e**)) curved and predefines the curvature of the chamber. In contrast, the course of other organic incorporations (yellow arrows in (**d**, **e**)) is wavy. The blue arrow in (**b**) and (**e**) indicates the cortex. The thickness of the latter varies slightly between 2–4 µm (**e**).

**Figure 2:**
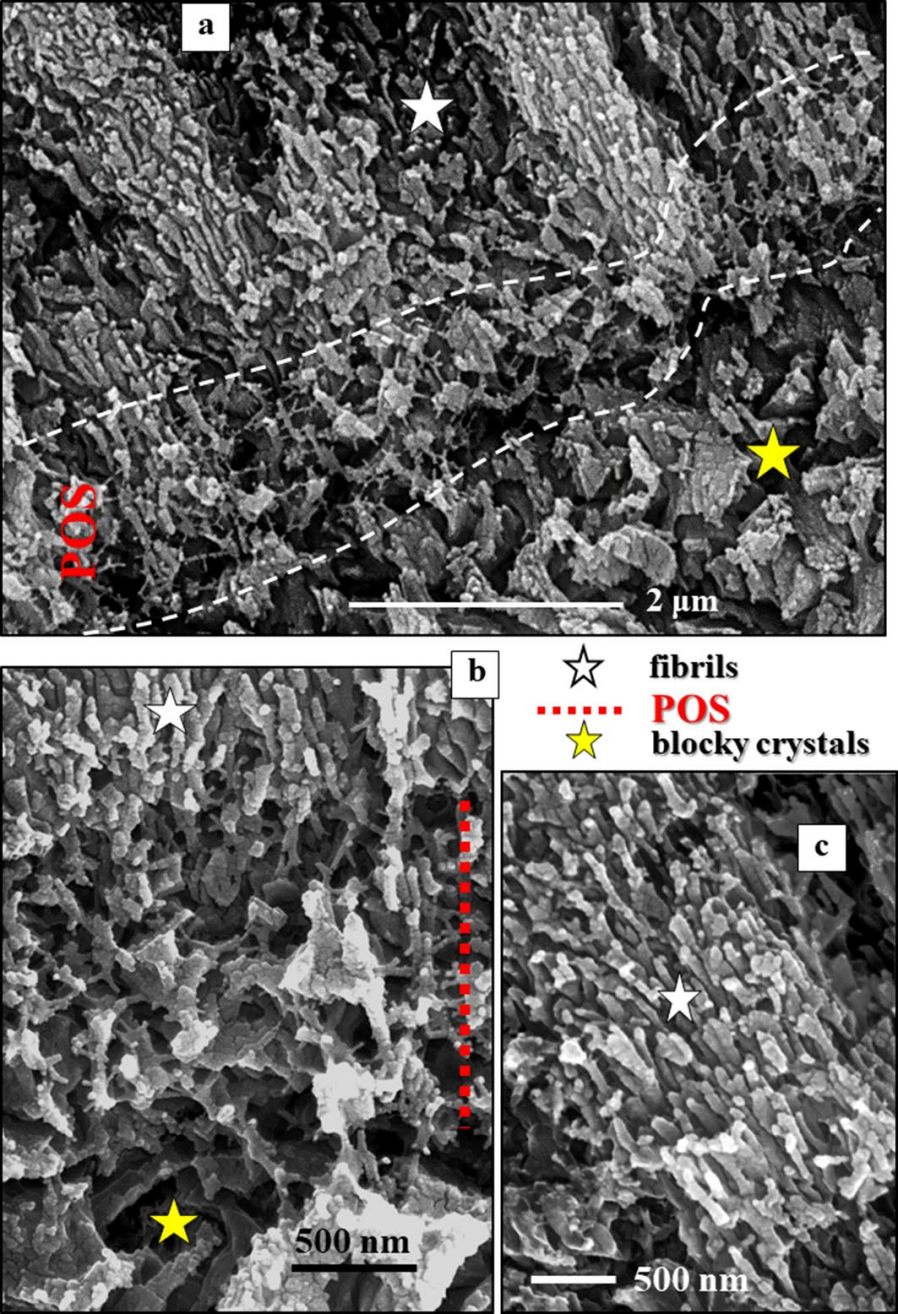
BSE images of shell cross-sections depicting the site of the primary organic sheet (POS, **a**, **b**) as well as the two microstructures that comprise the shell of *Pulleniatina obliquiloculata*: blocky crystals (yellow star in (**a**, **b**)) within and below the POS, and nanofibrils (white star in (**a**, **b**, **c**)) above the POS. See also Fig. S2. The nanofibrils nucleate within the POS; clusters of parallel nanofibrils form elongated, sheaf-like, crystals (white star in (**c**)). The latter crystals form most of the *outer shell wall*. The blocky crystals form the entire *inner shell wall*, the layer below the POS*.*

**Figure 3:**
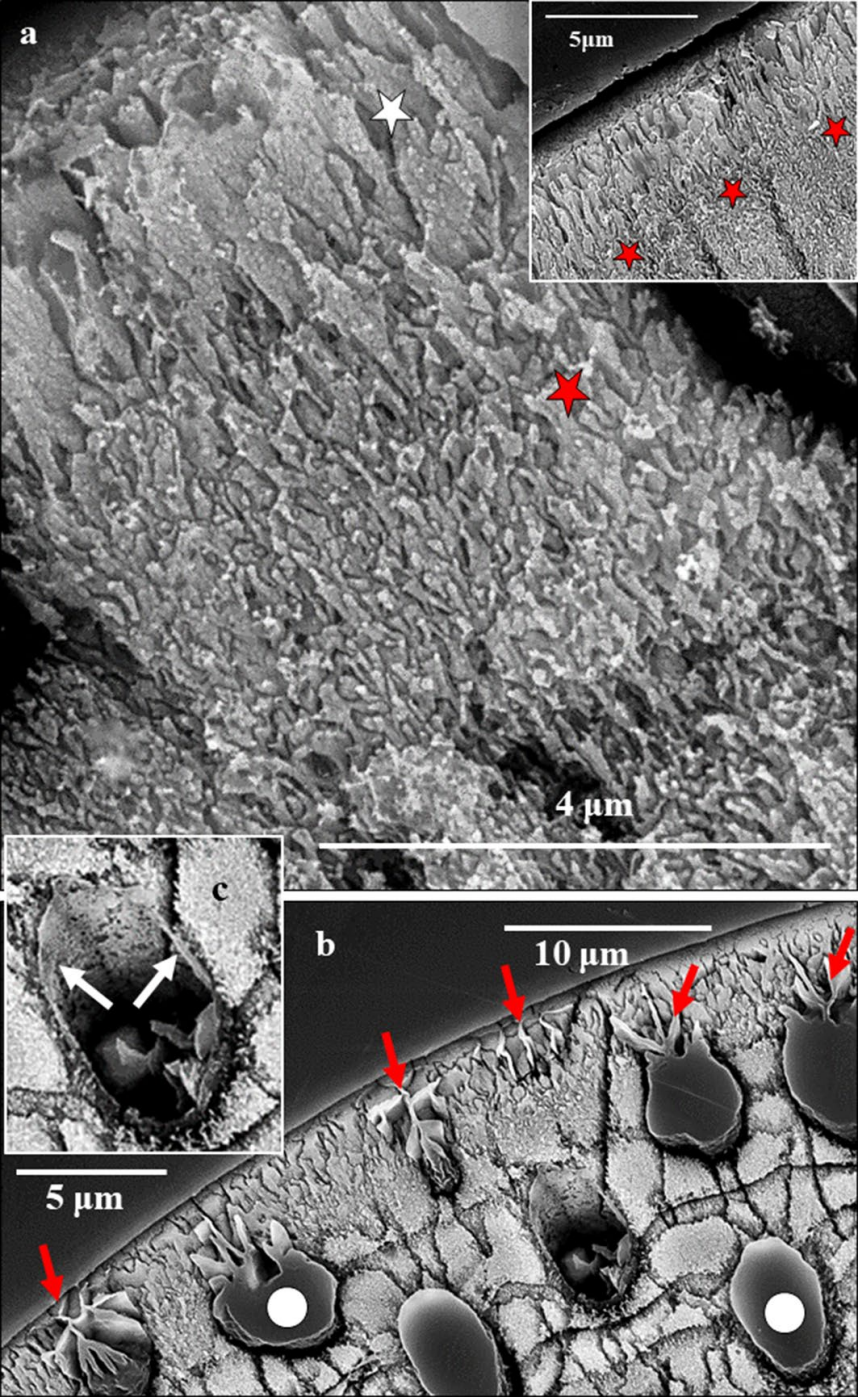
BSE images of cross-sections through the shell of *Pulleniatina obliquiloculata* visualizing the enlargement of crystal size towards outer shell surfaces. (**a**) development of irregularly shaped, platy crystals from the nanofibrils at/within the POS. Red stars in (**a**) and in insert in (**a**) indicate shell layers formed of fused elongated/sheaf-like crystals. Assemblies of large, plate/blade-shaped crystals comprise the cortex (white star in (**a**)). (**b**): At outermost shell surfaces, fissures and cavities are present between the large plates/blades. At sample preparation the fissures between the plates become infiltrated with the used low-viscosity EPON resin (red arrows in (**b**)). White dots in (**b**) indicate pores filled with EPON resin. White arrows in (**c**) point to the biopolymer film that lines the inner surface of a pore. See also Fig. S3.

In sagittal sections through the shell wall of *Pulletiniatina obliquiloculata* we observe two layers formed of crystals with distinct morphologies (Figs. 1, S1, S2). These are separated from each other by a network of biopolymer fibrils (Figs. 1, 2). We consider the latter to be remnants of the Primary Organic Sheet (POS). Outermost shell layers consist of an aligned array of fibril to platy crystals (Figs. 1, 3, S2), innermost shell layers are formed of blocky crystals (Figs. 1, 3, S2).

**Figure 4:**
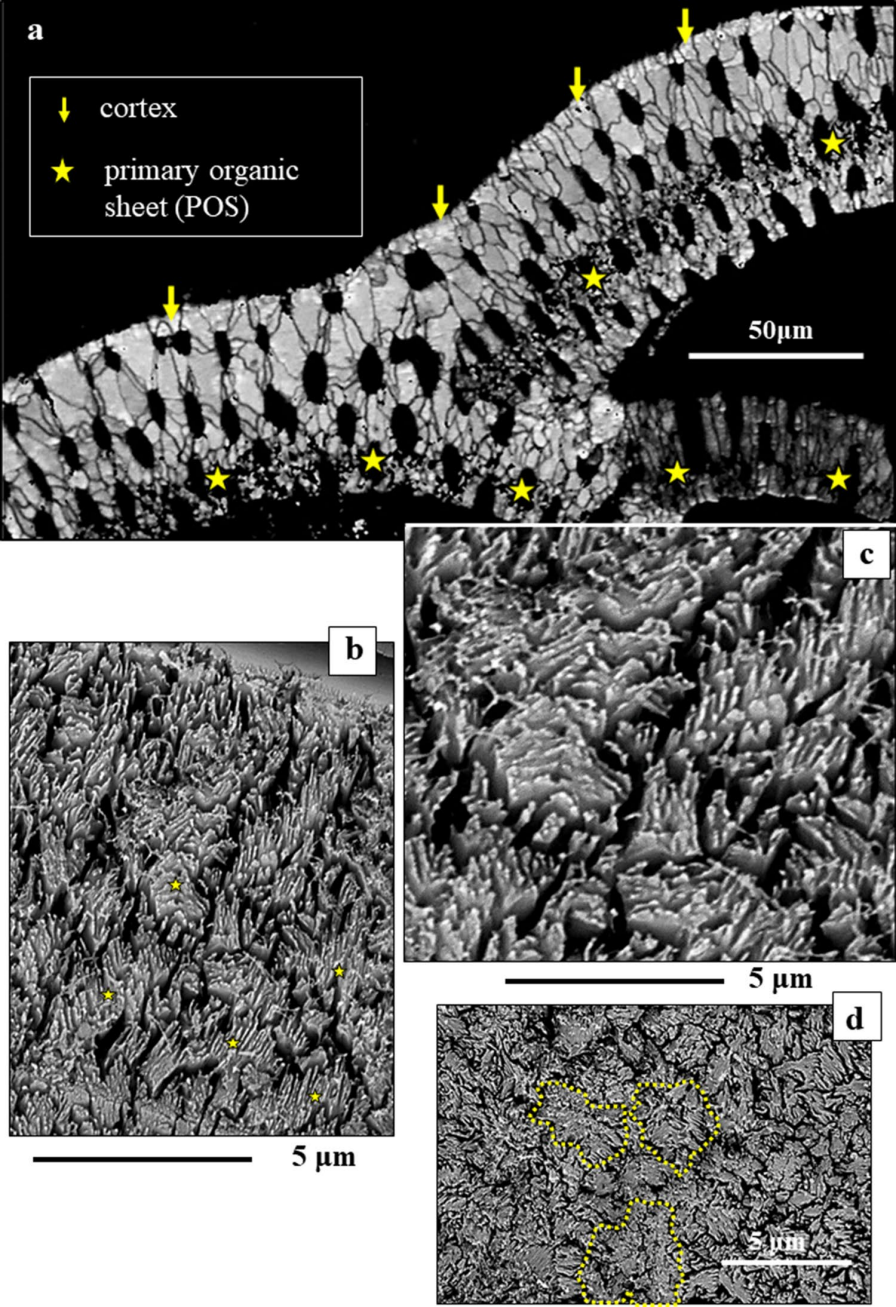
Tangentially oriented cut through the shell wall of *Pulleniatina obliquiloculata* (**a**) allowing view from above, onto the surface of the cone-shaped crystal units ((**b**)–(**d**)). The cone-shaped crystals are internally structured (**b**, **c**) and intertwine (**d**). (**a**) EBSD band contrast measurement in grey; (**b**, **c**, **d**) BSE images of etched surfaces. Yellow stars in (**a**) indicate the course of the POS, the latter is well-visible through the accumulation of small crystallites. Yellow arrows in (**a**) point to the cortex. Yellow dashed lines in (**d**) mark the outlines of the tangential surface of three cone-shaped crystals.

*P. obliquiloculata* forms porous shells (Figs. 1, 3, 4). The inner surface of the pores is lined by an organic membrane (Figs. 3c, S3). For the analytical techniques used in this study the shells had to be embedded in low-viscosity EPON resin. The latter infiltrated the pores (dots in Figs. 1b,c, 3b). In tangential cuts through the shell we see that the outer shell section is formed of crystal units with sizes increasing outward from the POS (Fig. 4a). Tangential views onto these units disclose their internal structure (Fig. 4) and interdigitating dendritic-fractal morphology (Fig. 8).

The organic network of the POS separates the two shell layers (Fig. 2). The POS templates nucleation and the start of calcite growth^21^. As visualized in Fig. 1, the course of the POS is even (Fig. 1e,f), in contrast to other organic intercalations into the shell that have an undulating course (Fig. 1e,d). We consider the latter organic material as a remnant of the Outer Organic Lining (OOL), secreted at the end of the sequential cycles of chamber formation^21^. *P. obliquiloculata* shells show a zonation in sulfur and magnesium (Fig. S4), which are positively correlated (Fig. S4). Sulfur is indicative of organic substance, hence, zones with an increased sulfur content point to the organic linings that cover outer and inner shell surfaces. As at each new chamber formation event older chambers become covered with an additional layer of calcite, at each shell growth stage the OOL that covers the outer surface of the shell becomes incorporated into the shell (Fig. 1e). We did not observe such episodic intercalations of organic substance into inner shell layers, between the POS and inner shell surface (Fig. 1e, f).

Crystals that form the shell have distinct morphologies:

1. In the *inner shell layers* (between the POS and the inner shell surface) we find *blocky crystals*. The morphology of the blocky crystals does not change between the POS and the inner shell surface (Figs. 2a, S2).
2. Most of the shell (the layers between the POS and the outer shell surface) is formed of crystals which initially have a *nanofibrillar* structure. The calcite nanofibrils develop within the POS and have diameters of about 50 nm reaching lengths of 1 μm or beyond (Fig. 2c, 3a).
3. Further away from the POS, towards outer shell surfaces, the dimension of the nanofibrils increases, their shape becomes platier. The fibrils evolve into *platy entities* (Figs. 3a, S3). These nanoplates have thicknesses in the 50–100 nm range (Fig. 4b–d) and lateral extensions in the order of 1 μm (Figs. 3, 4, S3).
4. Close to the cortex the, now, *platy crystals fuse and form a compact layer of calcite* (Figs. 3a, S2).
5. Of this compact layer *large plate/blade-shaped crystals* evolve (Fig. 3a); arrays of these plates/blades *form the cortex*. These plate/blade-shaped crystals are stacked in parallel and have ragged, dendritic morphologies. At embedding in EPON the fissures between the platy crystals of the cortex become infiltrated with resin (red arrows in Fig. 3b).

**Figure 5:**
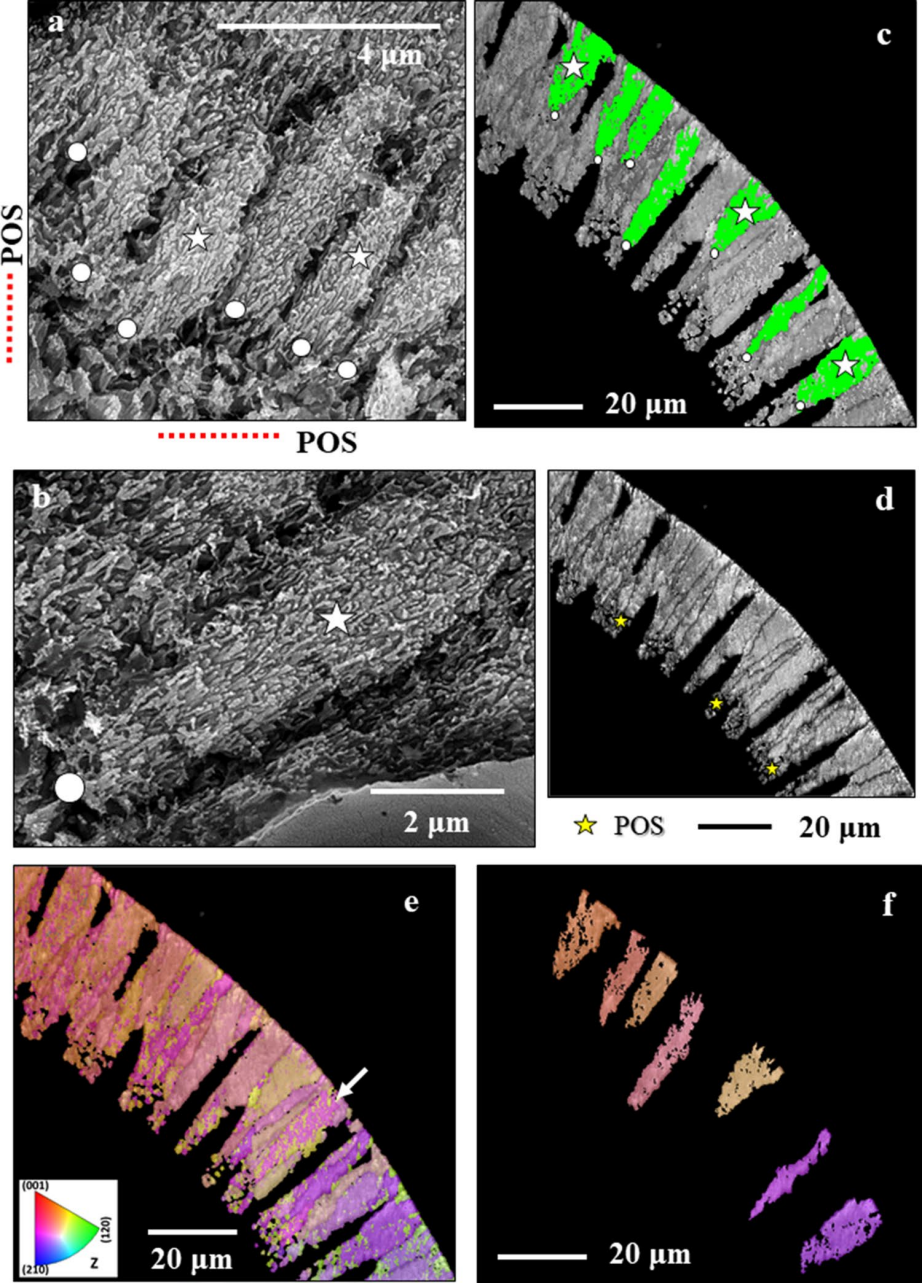
The crystals and crystal units in the shell of *Pulleniatina obliquiloculata.* (**a**) and (**b**) BSE images of elongated, sheaf-like crystals (white stars). These start to form at the POS (white dots in (**a**) and (**b**)) and comprise most of the cone-shaped calcite entities (**c**), (**d**), (**f**). (**c**) and (**d**) EBSD band contrast images in grey visualizing the extent of the cone-shaped units; (**c**) highlighted in green are some, arbitrarily chosen, cone-shaped crystals. (**e**), (**f**) calcite crystal orientation obtained from EBSD, for (**e**) an entire shell wall section and (**f**) the selected cone-shaped units that are shown in (**c**). From the uniformity in color it is evident that the calcite within the cone-shaped units have uniform crystallographic lattice orientation.

In contrast to inner shell layers, for the outer shell wall, which grows in thickness with every new chamber formation cycle, we observe a tremendous but continuous change in crystal size, morphology and internal nanostructure. The calcite nanofibrils form bundles or sheafs in which the crystallographic lattice orientation of all fibrils is the same (EBSD results, Fig. 5e,f). Their lattice orientation continues into the nanoplates/plates, up to the cortex (Fig. 5e,f). Hence, we see *elongated mesocrystal units with coherent lattice orientation* (Figs. 5, 11), however, *with an evolving internal nanostructure* (Figs. 3a, 9). The above described crystal morphologies are not just singular or surface features. We sectioned *P. obliquiloculata* shells at different depths and always observed the above described crystal size and morphology characteristics (Fig. S5). The evolution from nanofibrils via nanoplates to plates and blades results in a denser mineralization from inside to the outside, relative to the shell material formed of the blocky crystals between the POS and inner shell surface (Figs. 3, S2).

On sagittal cross-sections through the shell wall, *cone-shaped calcite crystal units* with lengths up to 20 μm and widths up to 10 μm become apparent in EBSD measurements (Fig. 5). These crystal units start to grow from one spot, in general, from or close to the POS, and widen towards outer shell surface. Note that these conical calcite crystal units with tens of micrometers in size, that we see with EBSD (Fig. 5c–f), have the internal nanostructure shown in Figs. 2, 3, 4, S1, S2. Within such a conical crystal unit there is a smooth and steady transformation of internal structure from nanofibrils, via bundles of fibrils to plate/blade-shaped domains of nanoscale thickness but microscale lateral extension.

**Figure 6:**
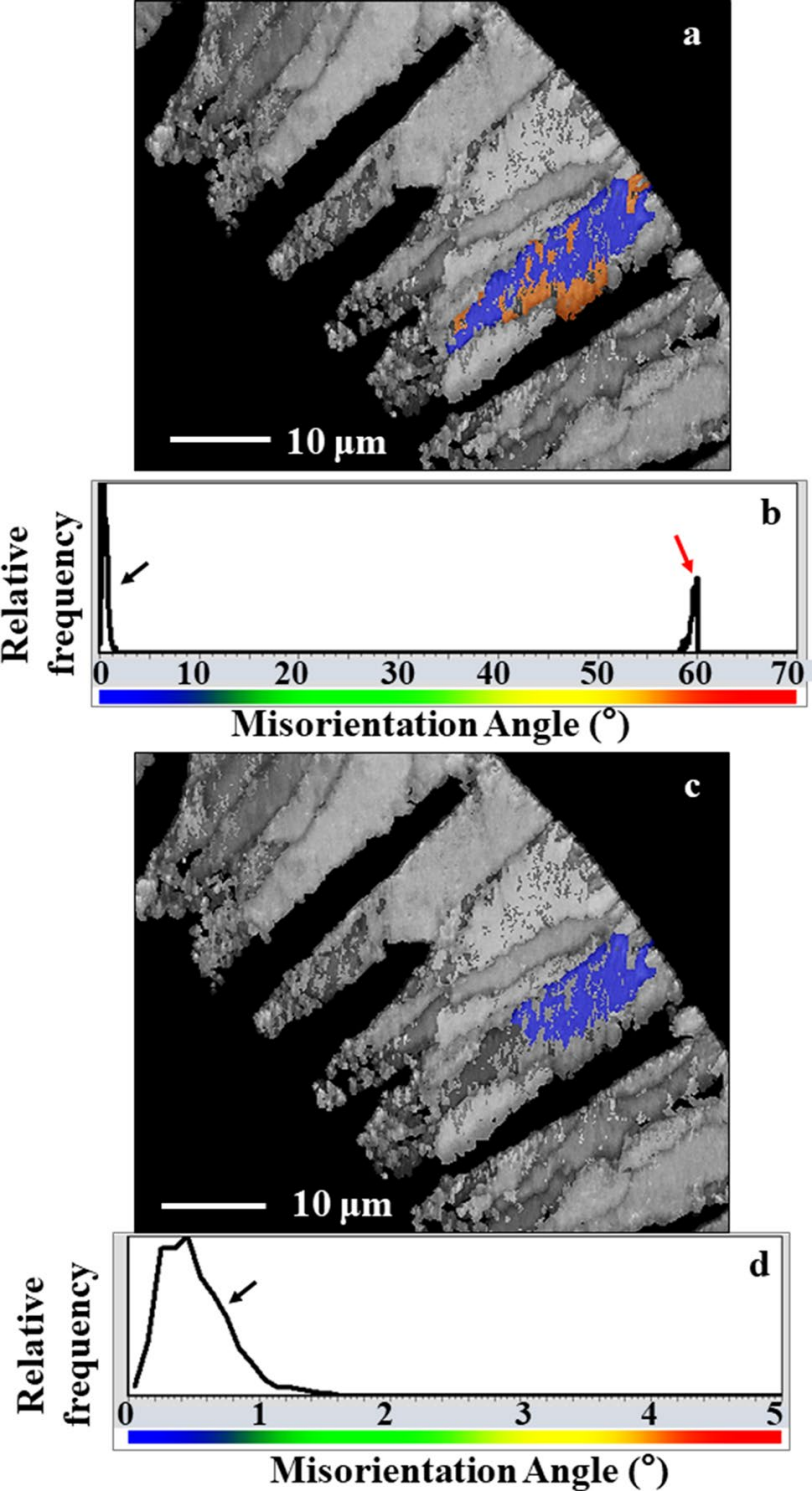
Twinned crystals and twin individuals/domains in *Pulleniatina obliquiloculata* shells. (**a**), (**c**) EBSD band contrast measurement (given in grey) obtained on a cross-section through the shell wall. In color in (**a**) and (**c**) are the corresponding individuals/domains of a twinned, cone-shaped, crystal unit. The twin individuals/domains are shown with blue and orange colors (**a**). These are misoriented to each other by 60°, as the two peaks (black and red arrows in (**b**)) in the relative frequency—misorientation angle diagram in (**b**) demonstrate. The two peaks in (**b**) are sharp, hence, misorientation of calcite crystallites within each of the twin individuals/domains is very low. (**d**): It is shown for one twin individual/domain that calcite crystallite misorientation is well below 1°. Hence, the calcite within each twin individual/domain is single-crystalline.

**Figure 7:**
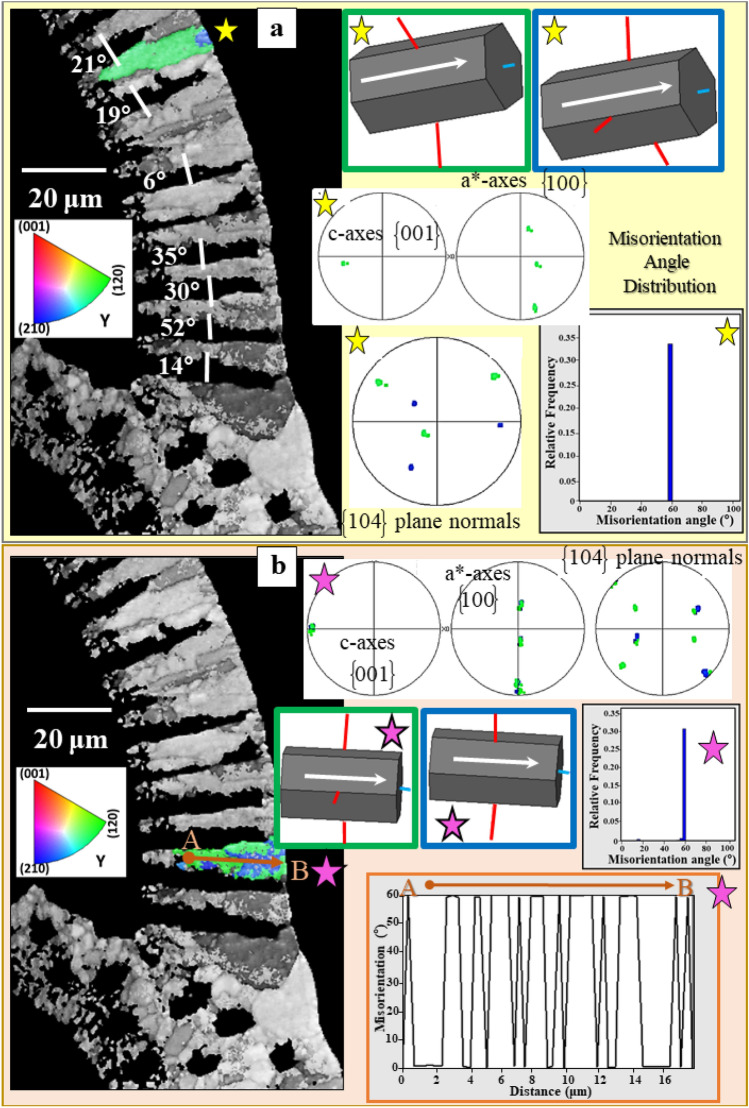
The different misorientations between calcite within and between adjacent cone-shaped units in *Pulleniatina obliquiloculata* shells. (**a**) and (**b**) EBSD band contrast measurement (given in grey) of a cross-section through the shell wall with, for a selected cone-shaped unit (yellow and magenta stars in (**a**) and (**b**)), mode of twin individual/domain orientation (sketched crystals), corresponding pole figures, misorientation angle distribution and misorientation-distance diagrams. The pole figures and latter diagrams demonstrate that the calcite of the cone-shaped units is twinned. Neighboring cone-shaped units are only misoriented to each other (**a**). Calcite c-axes are always perpendicular to the shell wall (white arrows in and orientation of the sketched crystals) and rotates with the curvature of the shell; see the slight change in calcite c-axis orientation of the sketched crystals.

Investigating EBSD data of the calcite crystal units in more detail, we find several orientation domains within them, shown in blue and orange colors in Fig. 6a. These domains are mutually related by a twin-orientation relationship of 60° rotation around the c-axes, i.e. around [001] (Fig. 6b). The frequency of these twins is reflected by the prominent peak at 60° in the misorientation angle distribution diagram (Fig. 7a,b). The calcite within each domain is single-crystal-like with a mosaic spread smaller than 0.5° (Fig. 6c,d).

Pole figures of such twinned cone-shaped crystal units (yellow or magenta star in Fig. 7a,b) indicate similar c- and a*- axes orientation, however, with the {104} poles being rotated by 60° around the [001] axis between the two domains (Fig. 7). The twin domains/individuals are not separated by a single interface plane, they are strongly interdigitating within a cone-shaped unit (Fig. 6a, the unit shown in Fig. 6a is indicated with a magenta star in Fig. 7b). Along a profile from A to B that crosses these interfaces, we observe recurrent switching between 0° and 60° in the misorientation-distance diagram (Fig. 7b). We find, for this particular example, 1.5 twin walls per micrometer, what equals to 1 twin wall every 0.7 µm. In summary, the {001}, {100}, and {104} pole figures, the prominent peak at 60° misorientation in the misorientation-angle-diagram, and the reoccuring 60° misorientation in the misorientation-distance-diagram indicate unequivocally that the calcite of the cone-shaped units is polysynthetically twinned.

Calcite c-axis is always perpendicular to chamber-wall surface and rotates with the curvature of the chamber wall (c-axis orientation of the sketched crystals in Fig. 7). Adjacent cone-shaped units are related by a small relative tilt of the c-axis following the curvature of the shell and random angles of relative rotation around <001> (Fig. 7).

**Figure 8:**
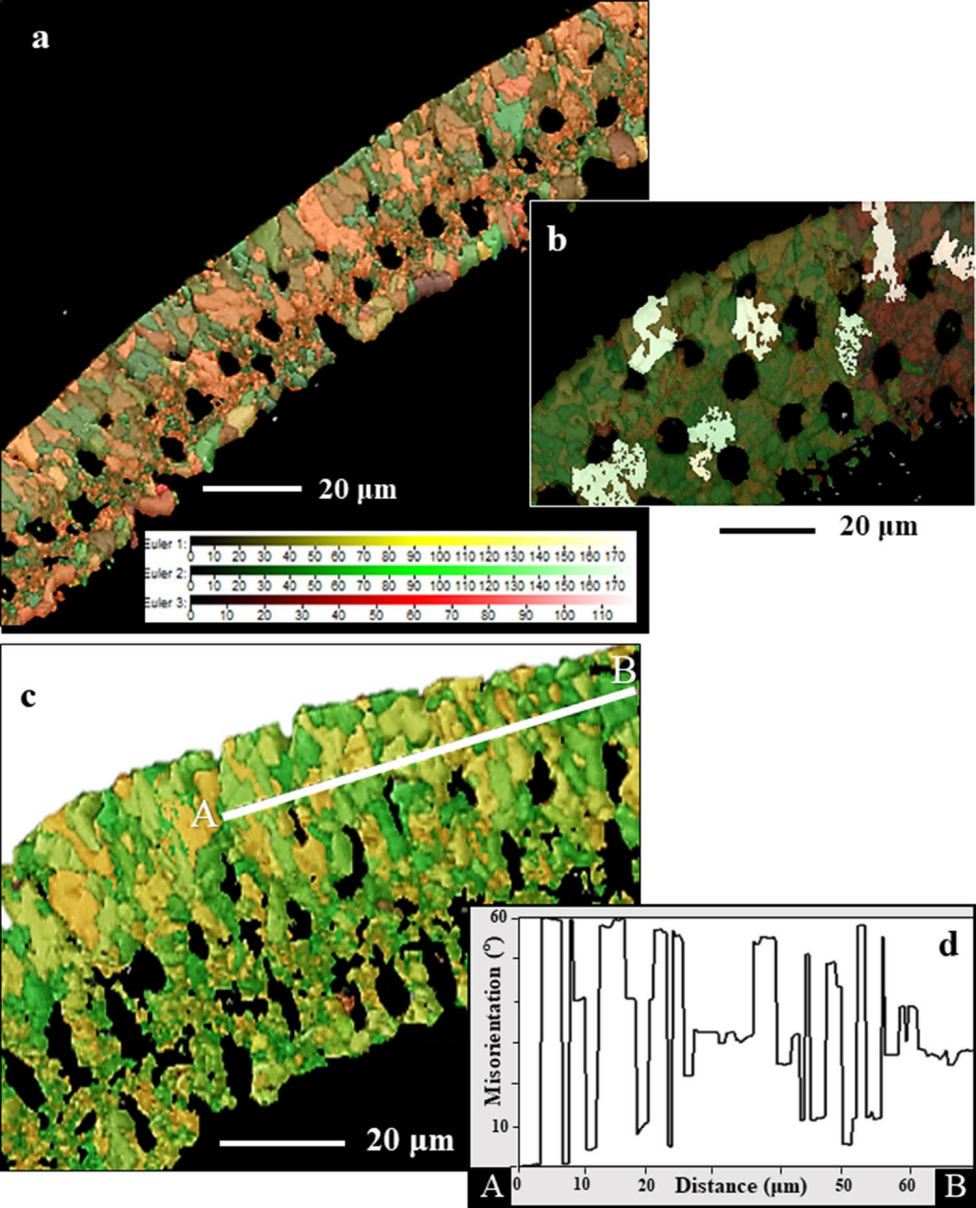
Tangential view onto the cone-shaped crystal units of *Pulleniatina obliquiloculata*. (**a**) and (**c**) EBSD scans with color coding for crystal orientation. Well-visible is the dendritic-fractal morphology of the cone-shaped crystals (**a**), (**b**), (**c**). Note the strong interdigitation of the cone-shaped entities (**a**), (**c**). The misorientation versus distance diagram (**d**) for the profile A to B (**c**) demonstrates that the cone-shaped units are misoriented relative to each other by random angles and are not related to each other by a twin relationship.

Tangential cuts through the shell allow views onto cone-shaped units (Figs. 8, S6). Well observable is the irregular, fractal-dendritic morphology of cone-shaped units (Fig. 8b), their large variation in size and their strong interdigitation (Fig. 8a,c). It is obvious that the twin relationship is within and not between adjacent cone-shaped units (misorientation-distance diagrams in Figs. 8d, S6).

## Discussion

### Morphogenetic and structural control of mineralization by the organic scaffold and the crystal growth process

A unique phenomenon that distinguishes foraminifera from other invertebrates with exoskeletons is that foraminifera extend, at each new chamber formation event, their cytoplasm beyond their skeleton. Foraminiferal cytoplasm is highly mobile, especially the cytoplasm that fills the penultimate and ultimate chambers, that forms the organic envelope of the ultimate chamber and extends over the external shell surface. The cytoplasm of foraminifera is always in bi-directional motion and ensures exchange and communication between external and internal environments^22^.

Foraminiferal cytoplasm is differentiated into (1) a compact endoplasm present in older chambers, (2) a reticulate endoplasm present mainly in the final chamber, and (3) a rhizopodial ectoplasm defining the outermost part of the final chamber and covering the entire shell surface^13,15^. The rhizopodial ectoplasm forms elongate extensions, the pseudopodia. Pseudopodia are projections of eucaryotic cell membranes and consist of networks of actin filaments^21,23^. On the basis of their shape, different types of pseudopodia are distinguished: lamellipodia, filopodia, reticulopodia and globopodia^23–25^.

Foraminiferal carbonate shell mineralization is a multi-stage process that is carried out and guided by different pseudopodial organizations^21,26^, however, always in combination with specific physiological controls that develop at active calcification^27–30^. Chamber formation starts with the emergence of the cytoplasm bulge, the globopodium. The globopodium has a granular, compact inner, and a less dense, translucent, outer portion^31^. An array of rhizopodial strands radiates from the surface of the globopodial bulge and forms a protective outer sphere^31–33^. Calcification starts at the periphery of the translucent section of the globopodium within a network of globopodial/rhizopodial filaments, the POS^21,31–33^. The globopodial stage of chamber formation is terminated with the complete mineralization of the rhizopodial, actin-enriched, filaments.

Our study shows that the globopodial/rhizopodial strands of the POS determine many structural characteristics of the first-secreted crystals: size, shape (Figs. 2, 5), crystallographic orientation (Fig. 11), and presence/absence of the 60°/[001] twin orientation (Figs. 10, 11). Within the POS network of *P. obliquiloculata* we find fibril-shaped and blocky crystals. Hence, the biopolymer substance of the POS is capable of templating both main types of crystals that form the shell: the *nanofibrils* that comprise the twinned, cone-shaped, units (Figs. 5, 6, 7) and the *blocky* crystals which are generally not twinned (Figs. 2, S2).

With ongoing mineralization, crystal deposition occurs also in direct contact with the POS. Hence, the organic template that guides nucleation and determines structural and crystallographic characteristics of the first crystals becomes embedded within shell wall carbonate and becomes sealed off from further exchange with the external environment. Its active function ends when the globopodial stage of shell formation is completed.

Concomitant to mineralization of the rhizopodial network of the POS, the globopodial strands transform to lamellipodia^21^. The latter consist of cytoplasm material and are reticulate lamellae that line inner- and outer shell surfaces^21,31–36^. Subsequent to mineralization of the POS network (globopodial stage of shell formation), further crystal growth is mediated by lamellipodia (lamellipodial stage of shell formation). Banner et al.^35^ were among the first to stress the importance of inner and outer organic linings for the determination of foraminiferal skeletal morphogenesis. Tyska et al.^34^ give an excellent summary of function, composition and the structural nature of foraminiferal organic linings. Combining the latter two studies and our work, we conclude that major structural characteristics of foraminiferal calcite are predetermined when the POS network becomes mineralized. Maintenance of these structural characteristics during shell wall growth on both sides of the POS is sustained through the function of lamellipodia and by an increase in intracellular pH^28–30^ and enhancement of supersaturation (this study).

**Figure 9:**
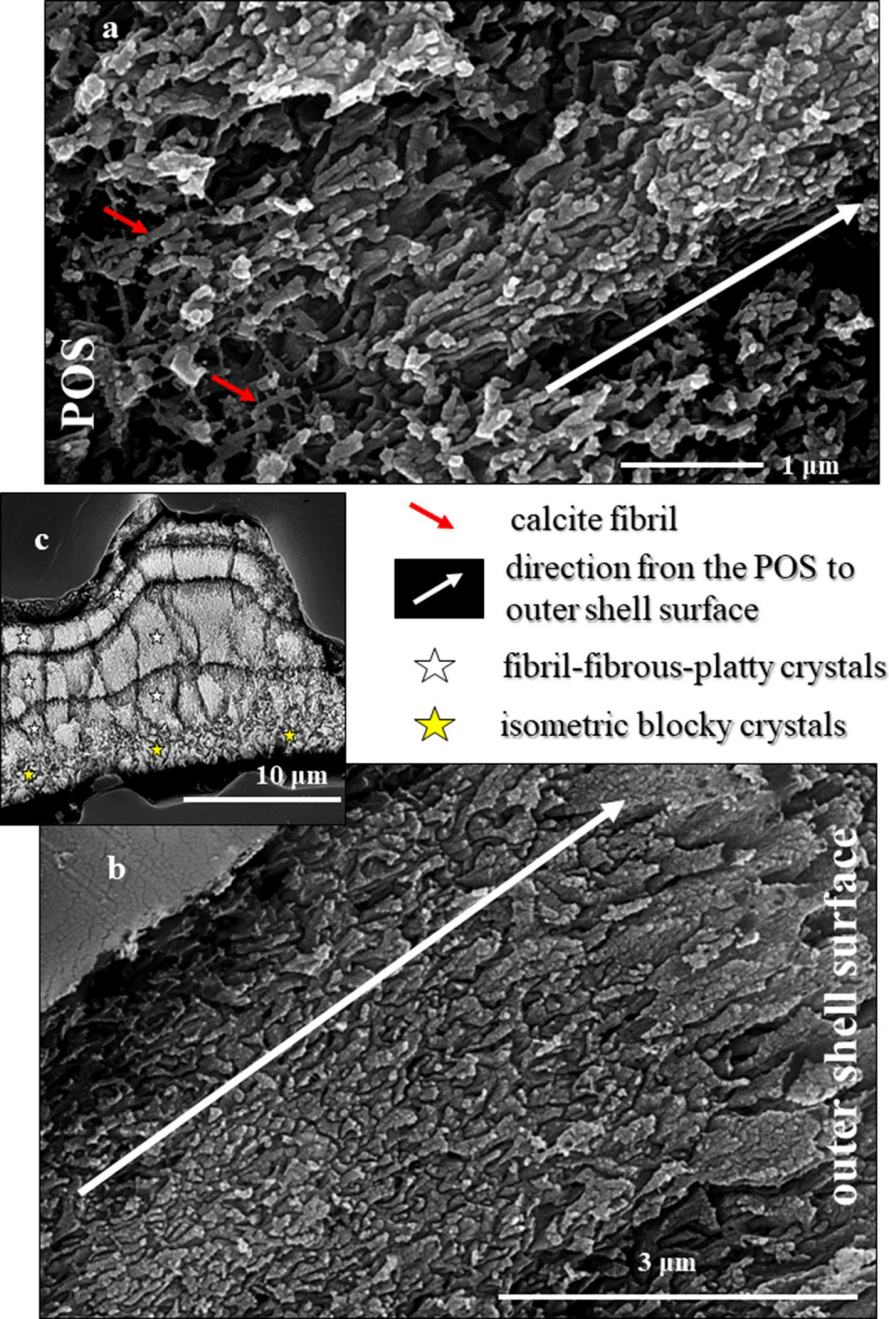
The smooth and steady transformation of crystal morphology and size within a cone-shaped twinned crystal unit; starting with the nanofibrils within the POS (red arrows in (**a**)) and developing to the large plate/blade-shaped crystals at outer shell surfaces (**b**). (**c**) Intercalations of organic substance related to successive chamber formation cycles (undulating black lines) do not interrupt the steady transformation of crystal shape and size, nor the twinning characteristic of the calcite within the cone-shaped units. (**a**), (**b**), (**c**) BSE images of an etched sagittal cross-section through the entire shell wall of *Pulleniatina obliquiloculata*.

Lamellipodia are dynamic and active^23,31,34^. Figures 1, S3, S4 visualize periodically occurring remnants of organic substance within the outer shell wall. For Rotaliida, at each new chamber formation event a new layer of calcite is deposited over the surface of all previously formed chambers^1,8^. We do not see signs of new crystal nucleation at this stage, but rather a smoothly continued growth of the pre-existing crystals over the entire shell wall (Figs. 9, S8), from the POS to outer shell surfaces. Being reticulate, the organic substance of the lamellipodia is neither a barrier to crystallization nor an additional template for additional nucleation; it serves as a guide for further smooth crystallization. Is crystallization resumed again, the mineral phase, the crystal orientation and the type of nanostructure are kept as they were for the crystals that were secreted during previous chamber formation events. This is a unique mineralization characteristic and quite distinct from what is observed for, e.g., bivalve or brachiopod shells^37–39^. In the latter, organic membranes are rather divisions between adjacent biocrystals and adjacent shell layers with different microstructures^37–39^. As, at each new chamber formation, the globopodial and lamellipodial secretion stages are repeated^21,22,32,34^, new outer and inner lamellipodia are secreted as well. These promote the continued growth of the type of crystals that are determined for each chamber only once, and by the rhizopodial strands of the POS. Hence, the lamellipodial organic substance is also actively directing foraminiferal carbonate crystallization: guiding the smooth transfer of morphological and nanostructural characteristics during crystal growth up to the outer surface of the shell. It is not known yet if there is a difference in chemical composition or/and structural characteristics between inner and outer lamellipodial linings^34,36^. The strong difference in microstructure and crystallographic characteristics of crystals at the two sides of the POS points to some structural/compositional difference of inner and outer lamellipodia.

**Figure 10:**
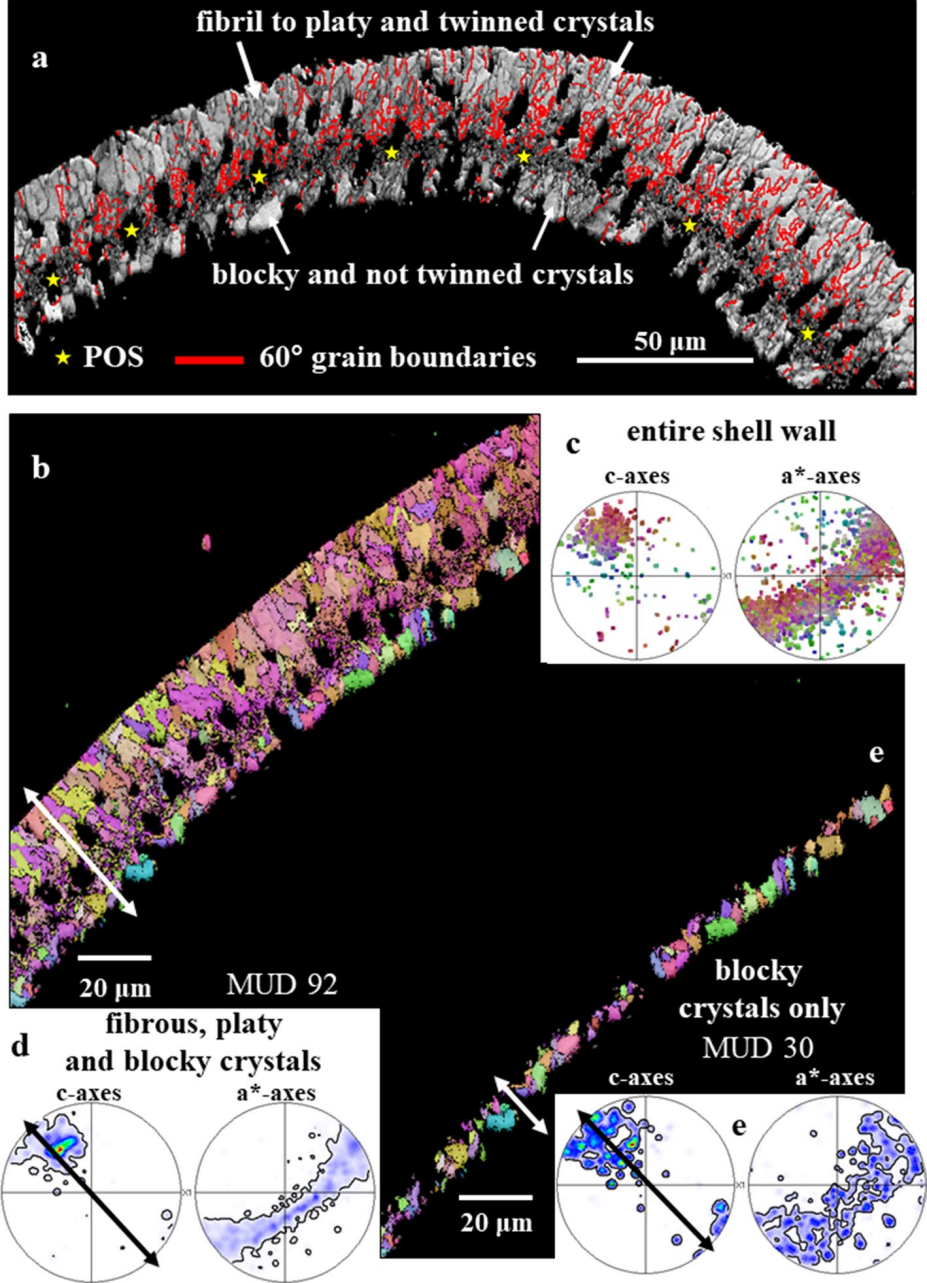
Structural characteristics of crystals and crystal assemblies on the two sides of the POS in *Pulleniatina obliquiloculata* shells. (**a**) EBSD band contrast measurement, with the distribution of 60° grain boundaries superimposed as red lines. (**b**) and (**e**) crystal orientation maps and (**d**) and (**f**) corresponding pole figures. Illustration of structural characteristics of the crystals and crystal units on the two sides of the POS: (1) fibril to platy crystal assemblages that are twinned (**b**) and (2) blocky, crystals that are not twinned (**e**). Blocky and fibril/fibrous-platy crystal assemblies have similar preferred orientation texture (**d**, **f**), they are, however, different in microstructure and crystal co-orientation strength. The blocky crystals are slightly less well co-oriented compared to the fibrous/platy crystals. Calcite c-axes are perpendicular to the shell surface for both the blocky as well as the fibril/platy crystal assemblages (white and black arrows in (**b**), (**d**), (**e**), (**f**)).

Crystallographic axes orientation is similar for the crystals deposited along the two sides of the POS (Fig. 10b–e). However, crystal co-orientation strength is lower for the blocky calcite that forms inner shell layers than for the calcite of the outer shell layers (Fig. 10d,e). In the latter calcite units are strongly interdigitating (Figs. 8a,c, 10b) and individual crystal units have a hierarchical internal structure (Fig. 4).

### The nanofibrillar and dentritic-fractal crystals

The morphology of rotaliid foraminiferal shell calcite (this study and Yin et al.^18^) is strikingly different from that of other biogenic carbonates. Instead of carbonate folia, laths, fibers, tablets, prisms, columns of mollusks or brachiopods (Figs. S9, S10), rotaliid foraminiferal shell crystals display erratic/irregular morphologies with dendritic-fractal outlines and a specific internal nano/microstructure. It is, in addition, remarkable how internal mesocrystal nanostructure transforms smoothly and steadily from a fibril to a plate/blade during growth of the rotaliid shell (Fig. 9), a feature that is widely absent in other marine organism’s biomineralization. However, common with brachiopods and mollusks is the fact that carbonate c-axis orientation is perpendicular to hard tissue surfaces (Figs. 7, 10).

The above mentioned structural characteristics indicate that the calcite, especially the crystals that comprise outer shell layers, forms through the process of *growth competition*.

When crystallization takes place through *competitive growth*, many crystals nucleate close to each other in random orientations, and, at growth, compete for space^40,41^. As crystal growth speed is anisotropic, the growth development of crystals is orientation-selective. The likelihood for a minute/small crystal to grow to large entities decreases with the departure of the crystal’s fastest growth vector from an orientation normal to the nucleation template. The result of the growth competition process is a strong decrease in the number of crystals as one moves away from the nucleation substrate, accompanied by an increase in crystal size and generation of a progressively stronger crystal co-orientation strength. At growth competition only those crystals that have their fastest growth axis perpendicular to the nucleation surface continue to grow from the point of nucleation to outer shell surfaces. For calcite the fastest growth axis is the c-axis. Figures 9 and S7 show examples of crystal formation obtained through growth competition and visualize the difference in microstructure for crystals having dendritic-fractal (*P. obliquiloculata*, Rotaliida, Fig. 9) and crystals having regular crystal morphologies (*Argonauta hians*, Cephalopoda, Fig. S7).

**Figure 11:**
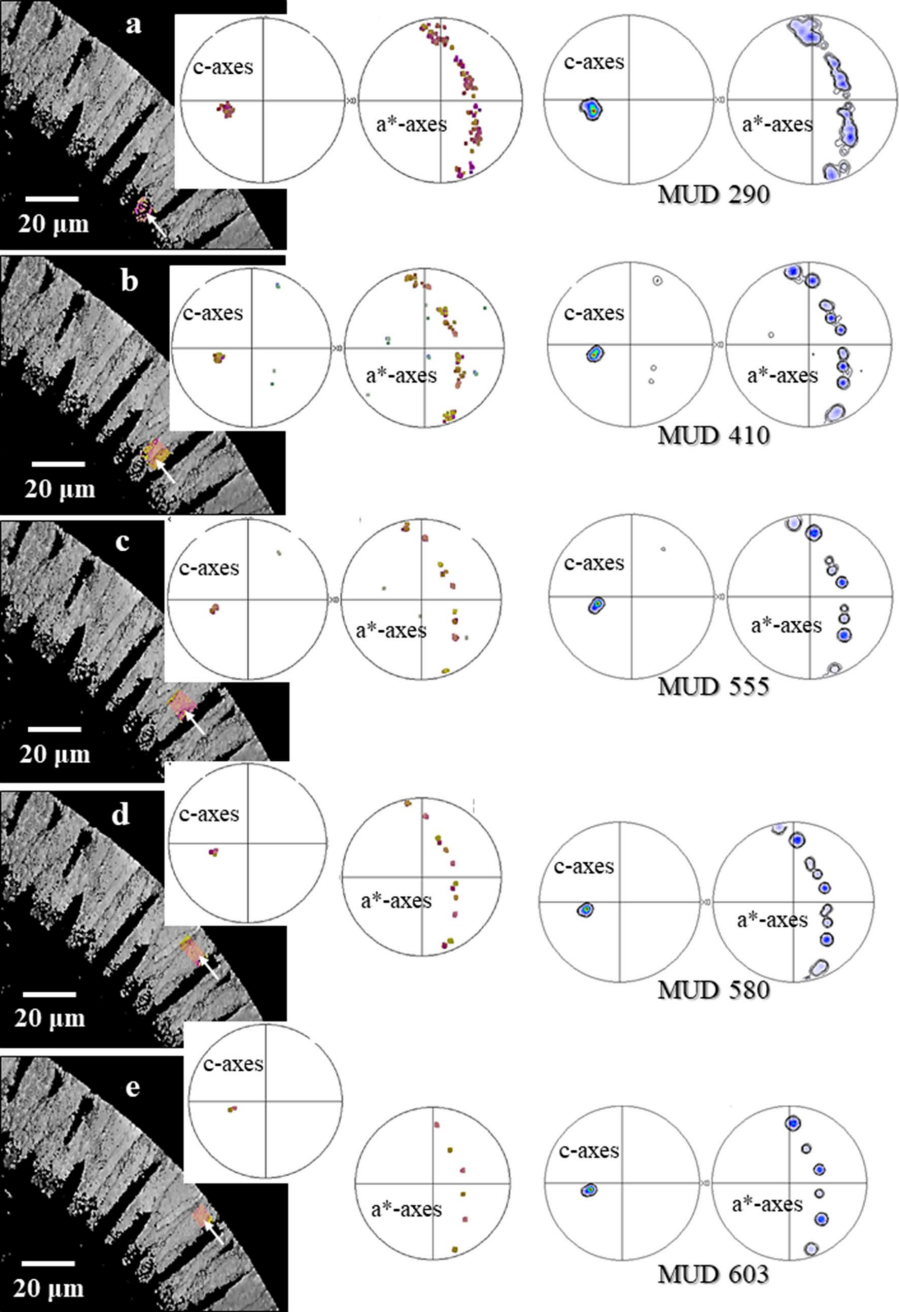
Development of crystal co-orientation strength from inner to outer shell wall layers on a cross-section through the shell wall of *Pulleniatina obliquiloculata*. (**a**)–(**e**) The white arrows in the EBSD band contrast measurement images (in grey) indicate the relevant section of a cone-shaped unit (in color) that was taken as a subset. The corresponding pole figures, given in (**a**)–(**e**), show crystal orientation data of these subsets only. (**a**): Shell wall portion at the POS and the shell section that is formed of the blocky crystals; (**e**): Shell wall section close and at the cortex. Crystal co-orientation strength is given with the MUD value. We find: (1) an increase in MUD, accompanied by a decrease in amount of crystals from the POS/blocky crystals (**a**) to the cortex (**e**), (2) however, at keeping a similar/comparable texture pattern for all the subsets. This indicates that the crystallographic axes orientation of the crystals is already determined within the POS, by the polymer network of the POS and that the texture of the crystals is not only given and initiated by the competitive growth process.

Shell formation through growth competition is not only observed for Rotaliida. It has been reported for the columnar layer of brachiopod shells^37^, for cephalopod shells^42,43^ and for prismatic layers of euheterodont bivalves^44^. Structural characteristics for shell growth through growth competition are well observable for the calcite of *P. obliquiloculata*, for the shell layer between the POS and the outer shell surface. We see an increase in crystal size, the outermost shell layer of *P. obliquiloculata* is formed of large crystals. These are assembled in parallel and have their c-axis orientation perpendicular to the shell surface. Nonetheless, it is very important to keep in mind that for *P. obliquiloculata* calcite, the preferred c-axis orientation is already present after nucleation in the POS (Fig. 11). This points to crystallographic orientation texture promoted also by the organic template, at first crystallization.

When crystals grow from solution, s*upersaturation* is the driving force for nucleation and growth^45–47^. At high supersaturation, nucleation is a very rapid, a catastrophic event, resulting in numerous, randomly oriented, nucleii^45^. We, indeed, have indications that *P. obliquiloculata* calcite is formed at high supersaturation: (1) shell formation is very fast; a new chamber is completed within about 6 hours^22,48^, (2) physiological results point to an increase in pH at mineral secretion^28–30^, (3) we find extensive formation of growth twins at and close to the POS (Fig. 10a).

On the basis of the *degree of supersaturation*, it is possible to define crystal growth regimes; these induce differences in crystal morphologies^49,50^. Crystals that form at *low supersaturation* and high mobility of ions on the growth surface, develop *well-defined morphologies* and smooth rational surfaces. An *increase in supersaturation* and decrease of ion mobility induces the *formation of dendritic morphologies*, while very high levels of supersaturation induce the *formation of spherulites* by the accumulation of stresses and secondary nucleation^50^. Dendritic to spherulitic crystal morphologies become developed at diffusive and continuous growth processes^50–52^; crystal growth processes that are dominant at high supersaturation. In analogy to^49,51,53^, we can conclude that the dendritic crystal morphologies indicate that *P. obliquiloculata* crystals form under increased/high supersaturation conditions and at reduced surface mobility.

Increasing pH increases the supersaturation for CaCO_3_ in the solution^54^. It is known that oscillations in pH drive the crystallizing system from super- to undersaturated states, from dissolution at low pH to rapid precipitation at high pH^51,52^. An increase in intracellular pH from 7, 7.5 to 9 is reported at foraminiferal shell calcification^30,48^. The high pH/high supersaturation conditions lead to the formation of dendritic crystal morphologies of rotaliid shell calcite as well as to the high precipitation rates. The organic matrix in which growth takes place limits ion mobility to channels through these organic linings and reduces mobility of ions on the growth surface. This translates into the formation of calcite nanofibrils/nanoplates and the fractal microscale crystal morphology. The accompanying competitive growth process ensures that of the many crystals that nucleate at the start of shell mineralization (due to high pH, high supersaturation), only few crystals grow to large entities and have preferred crystallographic axes orientation. A preferred orientation of c-axes perpendicular to shell surface is important for mechanical purposes. As calcite crystals cleave easily on their {104} planes, and the <001> or c-axis direction forms the largest possible angle with all {104} cleavage planes, potential cleavage cracks are prevented from going perpendicular to the shell. They are further deflected at each crystal grain boundary to neighboring crystals, which have random orientations around the c-axis. Thus, the axial/cylindrical texture provides a good solution to prevent fracture of the shell by calcite cleavage.

On the basis of our microstructure, texture results we can deduce that *P. obliquiloculata* calcite formation is the result of a combination of (1) strictly biological and (2) general physicochemical controls, the latter also found in the inorganic world. De Nooijer et al., Geerken et al.^10,48^ and Toyofuku et al.^29^ report that the start of calcite formation is the result of an increase in pH at the site of calcification, triggered by exchange of Ca^2+^ for protons. Nucleation starts within the rhizopodial network of the POS. Crystallographic and morphological characteristics of the first-formed crystals are determined by the biopolymer strands of the POS. Thus, first crystal formation is mainly controlled by *biological determinants:* (1) physiological processes that influence nucleation and (2) the templating effect of the biopolymer network of the POS. Continuation of calcite crystal growth, transmission of structural characteristics, such as crystal size, shape, orientation/misorientation, the twinning pattern is, to a large degree, regulated by *physical, thermodynamic and kinetic effects:* the supersaturation condition and the *competitive growth process*. Hence, subsequent to mineralization of the POS, further shell wall formation is determined mainly by physical processes, nevertheless, also guided by biopolymers, the lamellipodial linings.

## The 60°-{001} calcite twins and their implication

In addition to the nanofibrillar and dendritic-fractal crystal morphologies, the high accumulation of 60°/[001] calcite twins is the other outstanding structural characteristic that we observe for *P. obliquiloculata* shells (Figs. 6, 7, 10a). The twinned crystals have nanofibrillar/nanoplaty microstructure; blocky crystal assemblages are rarely twinned.

When based on genesis, twins are divided into growth, transformation and deformation twins^55–57^. Twins are defined as a regular intergrowth of two or more crystals/domains of the same phase, which are related to each other through a well-defined crystallographic rational orientational relationship^56^. In a twinned crystal the lattice orientation of one domain (twin individual) bears a definite rational relationship to the crystallographic orientation of the other domain (twin individual).

The twins in *P. obliquiloculata* shells are growth twins; they are not caused through mechanical deformation or phase transformation. Growth twins form by departures from a steady state during growth, their formation occurs under increased supersaturation^55,56^. As the highest degree of supersaturation is before or/and at nucleation, growth twins may form in the very first stages of the crystallization process. We observe that the highest accumulation of 60° grain boundaries, demonstrating the formation of 60°/[001] calcite twins, are within or/and at the POS (Fig. 10a). The calcite of these shell portions consists of bundles of nanofibrils (Fig. 2). Hence, mostly twinned in *P. obliquiloculata* shells are the nanofibril/nanoplate arrangements. For mechanical reasons it is not favourable to form the entire shell of fibrils. It is rather important that calcite c-axes become oriented perpendicular to the shell wall. The competitive growth process regulates the latter characteristic, initiates the formation of large, highly interlinked crystal units with their c-axes being normal to the surface of the shell.

In summary, our structural-crystallographic study of *P. obliquiloculata* calcite confirms the state of elevated pH^28–30^ at foraminiferal calcite biomineralization. The strong twin formation and the strongly prevailing highly irregular crystal morphologies indicate high supersaturation conditions at nucleation and first crystal formation, especially, at formation of the fibril crystals. The distinctive difference in crystal shape on the two sides of the POS might point to differences in supersaturation conditions.

Despite decades of research, there is still no general consensus on the process/processes that lead to foraminiferal shell biomineralization^10,58–61^. Multiple mechanisms are suggested: endocytosis of seawater, utilization of transmembrane ion-transporters, formation of ion-specific organic templates, generation of privileged space in conjunction to mitochondrial activity^10^. As supersaturation and pH are positively correlated for crystallization from solution^53^, at least one process that leads to foraminiferal calcite formation, namely, the increase in pH at the site of calcification^29,30,48^ is substantiated now from structural-crystallographic (this study) as well as from physiological-chemical^28,48^ observations, measurements and conclusions.

## Conclusions

While crystallographic preferred orientation of rotaliid foraminiferal calcite follows patterns frequently seen for other carbonate biomaterials and biomimetic analogues^62–65^, the nano- and microstructural characteristics are unique. For *P. obliquiloculata*, Rotaliida, we deduce the following conclusions:

1. The shell of *P. obliquiloculata* is formed of two main types of mesocrystals. These differ in size, morphology and crystallographic characteristics: blocky crystals, reaching 10 μm size, at inner shell layer below the POS, and fractal/dendritic mesocrystals, reaching tens of micrometers, having an internal structure that evolves smoothly from nanofibrillar to platy, at outer shell layers.
2. Within the biopolymer network of the POS that templates nucleation and first crystal growth we find both types of crystals.
3. The nanofibrillar/nanoplaty/platy calcite of outer shell layers is strongly twinned, according to the 60°/[001] twin relationship.
4. The dendritic-fractal morphology of the crystals and the strongly developed twinning indicates high supersaturation and high pH conditions at crystal nucleation and shell growth. Our structural results support the conclusions of^28,30,48^.
5. Crystals, especially those that form outer shell sections, form and grow through growth competition.
6. Calcite c-axes are perpendicular to outer and inner shell wall surfaces and rotate with the curvature of the shell vault. The latter may be a consequence of the competitive growth mechanism. However, the texture may also be promoted already at the stage of nucleation by the organic template of the POS.
7. Two determinants lead the fabrication of foraminiferal shell calcite, a mainly biological and a mainly physical determinant.

## Materials

Species of *Pulleniatina obliquiloculata* were obtained from sedimentary traps from the Eastern Pacific.

### Sample preparation

#### Sample preparation for etching, fixing and imaging with an FE-SEM

To visualize small-scale internal structures and the presence of organic material within the shells, shell cross-sections were etched. First, flat surfaces were obtained by cutting and polishing the shells with glass and diamond knives. The flat surfaces were then etched with a 0.1 M HEPES buffer (pH = 6.5) and 2.5% glutaraldehyde solutions for 90 and 120 s, respectively. Etching was terminated by rinsing the samples three times in 100% isopropanol for 10 s each. Subsequently, samples were critical-point dried and were coated with 4–6 nm of Pt/Pd. SE or/and BSE images were taken at 4 kV, with a Hitachi SU5000 FE-SEM. Images presented in this study show BSE contrast. SEM micographs were taken with a Hitachi SU5000 back-scatter detector and show BSE (backscattered electron) contrast. See also^62,63^.

#### Sample preparation for EBSD measurements

Foraminifera shells were embedded in low-viscosity EPON resin and were cut and polished with glass, trimming and diamond knives in an ultramicrotome. Specimens were oriented with the primary apertures positioned towards the knife. For EBSD measurements the samples were coated with 4–6 nm of carbon. Measurements were carried out with a Hitachi SU5000 field emission SEM, equipped with an Oxford EBSD and EDS detectors.

### Methods

#### Electron Backscattered Diffraction (EBSD) and Energy Dispersive Spectroscopy (EDS) measurements

EBSD measurements were performed with step increments between 100 and 200 nm. Calcite microstructure is shown with coloured EBSD maps, where similar colours visualize similar crystal orientations, while different colours indicate differences in crystal orientation. The term texture relates to the varieties of crystal orientations within a material and is given with pole figures. EBSD measurements were performed on a Hitachi SU5000 FE-SEM equipped with a Nordlys II EBSD detector. During measurements the SEM was operated at 15 and 20 kV, depending on the size of the crystallites. Data were collected and evaluated using the Oxford Instruments AZTEC and CHANNEL 5 HKL softwares. EDS measurements were carried out with an OXFORD X-Max80 EDS detector attached to a Hitachi SU5000 FE-SEM.

#### Terminology

*Microstructures* are presented with colour-coded as well as grey-scaled EBSD band contrast measurement maps and colour-coded EBSD orientation maps. The colouring code is indicated either in the figure or is stated in the relevant figure caption. In the crystal orientation maps, similar or distinct colours indicate similar or different crystallite orientations, respectively. *Band contrast measurement images* depict the signal strength in each measurement point. High signal strength corresponds to light grey colours and indicates strong diffraction at the crystal lattice. Faint grey or dark colours are indicative of non-diffracting substances, e.g. polymers, or of an overlap of minute crystallites that cannot be resolved (indexed) automatically with the EBSD software.

The *texture* (crystallographic preferred orientation) is presented with pole figures that give orientation data or the density distributions of these.

For the *density distributions*, we use the lowest possible setting for half width and cluster size in the CHANNEL 5 software: a half width of five and a cluster size of three degrees. The half width controls the extent of the spread of the poles over the surface of the projection sphere, a cluster comprises data with the same orientation.

Calcite crystallite *co-orientation strength (given with MUD values)* within as well as between the biocrystals and the biocrystal units is derived from density distributions of the measured EBSD data. The MUD (multiple of uniform (random) distribution) value is calculated with the Oxford Instruments CHANNEL 5 EBSD software. A high MUD indicates high crystal co-orientation strength, while low MUD values reflect a low strength of crystallite or/and mineral unit co-orientation. An MUD of 1 indicates random distribution and no preferred orientation, an MUD higher than 700 documents almost perfect co-orientation; single crystalinity^62–64^.

An *axial texture* is given when the c-axes show co-orientation (clustering in the pole figure around a single direction), while the corresponding a*-axes vary in orientation on a great circle perpendicular to the texture axis direction.

We describe the *phenomenon of twinning in biological calcite*. Twinning is an intergrowth of two crystals of the same phase. *A twinned crystal consists of twinned individuals/twinned domains.* The individuals/domains of a twinned crystal have different orientations; their orientational relationship is crystallographically defined through a mirror operation on a crystallographic lattice plane or rotation around a crystallographic axis. We prove a specific twin law with: (1) the presence and distribution pattern of, for a twin law, specific misorientation boundary, (2) for a twin law characteristic peak in the misorientation angle distribution diagram, and (3) pole figures for the twin law of calcite that we describe. Calcite has four twin laws and is twinned on the {001}, {012}, {018} and {104} twin planes, respectively. As the orientation relationship of twinned crystals has to be related through a mirror operation on a plane or rotation around an axis, e.g. when twinned according to the {001} twin law, common for the twin individuals/twin domains are the c- and a*-axes orientations. Hence, for the twinned individuals/twinned domains the c- and a*-axes orientations have to be similar, thus, orientation data have to fall on the same spot in c- and a*-axes pole figures. All the above described characteristics can be gained from EBSD measurements and have to be fulfilled for the definite proof of a twin relationship between adjacent crystals.

We describe in this contribution (1) calcite fibrils, (2) assemblies of fibrils to sheaf-like crystals, (3) fusion of sheaf-like crystals and, on a sagittal section through the shell wall, the formation of (4) cone-shaped calcite units/entities (Fig. S1). We show, in addition, the development of fibrils to irregularly shaped (5) platy crystals that seam outer shell surfaces. For definition of the above mentioned crystallites, crystals and calcite units and visualization of their morphologies see Fig. S1. One shell sample of *P. obliquiloculata* was sectioned and polished with a microtome in two different depth. The surfaces were subsequently etched, critical point dried and imaged with an SEM. We obtain on both sections very similar internal structures (Fig. S5). Hence, the fibrillar to platy nature of *P. obliquiloculata* calcite is a unique structural characteristic of this biomaterial.

Nucleation and crystal growth of Rotaliida foraminiferal calcite and aragonite occurs onto the biopolymer network of pseudopodial strands. These form the so-called Primary Organic Sheet, the POS.

## Supplementary Information

Supplementary Information.

## Data Availability

The datasets generated and/or analysed during the current study are available in the ZENODO repository. Web link: https://zenodo.org/record/6655936#.YqxO5HZBxaQ; https://doi.org/10.5281/zelando.6655936 as well as, on request, from the corresponding author.
