# Supplementary material for: The unique fibrilar to platy nano- and microstructure of twinned rotaliid foraminiferal shell calcite
Source: Sci Rep. 2023 Feb 7;13:2189. doi: 10.1038/s41598-022-25082-9 (PMC9905586; doi:10.1038/s41598-022-25082-9)
Supplement: Supplementary file 1 — Supplementary Information. [file 41598_2022_25082_MOESM1_ESM.docx]

**The unique fibrilar to platy nano- and microstructure of twinned rotaliid foraminiferal shell calcite**

J. Lastam^1^, E. Griesshaber^2^**^*^**, X. Yin^2^, U. Rupp3, I. Sánchez-Almazo^4^, M. Heß^5^, P. Walther^3^, A. Checa^6^, W. W. Schmahl^2^

1. Forschungszentrum Jülich, Institut für Energie und Klimaforschung, IEK-2, 52425, Jülich, Germany
2. Department für Geo- und Umweltwissenschaften, Ludwig-Maximilians-Universität München, 80333 Munich, Germany
3. Zentrale Einrichtung Elektronenmikroskopie, Universität Ulm, 89081 Ulm, Germany
4. Centro de Instrumentación Científica, Universidad de Granada, 18071 Granada, Spain
5. Biozentrum LMU München, 82152 Planegg-Martinsried, Germany
6. Departamento de Estratigrafía y Paleontología, Universidad de Granada, 18071 Granada, Spain, and Instituto Andaluz de Ciencias de la Tierra, CSIC-Universidad de Granada, Armilla, 18100, Spain

* Corresponding author: E. Griesshaber

**Supplementary Figures**

**
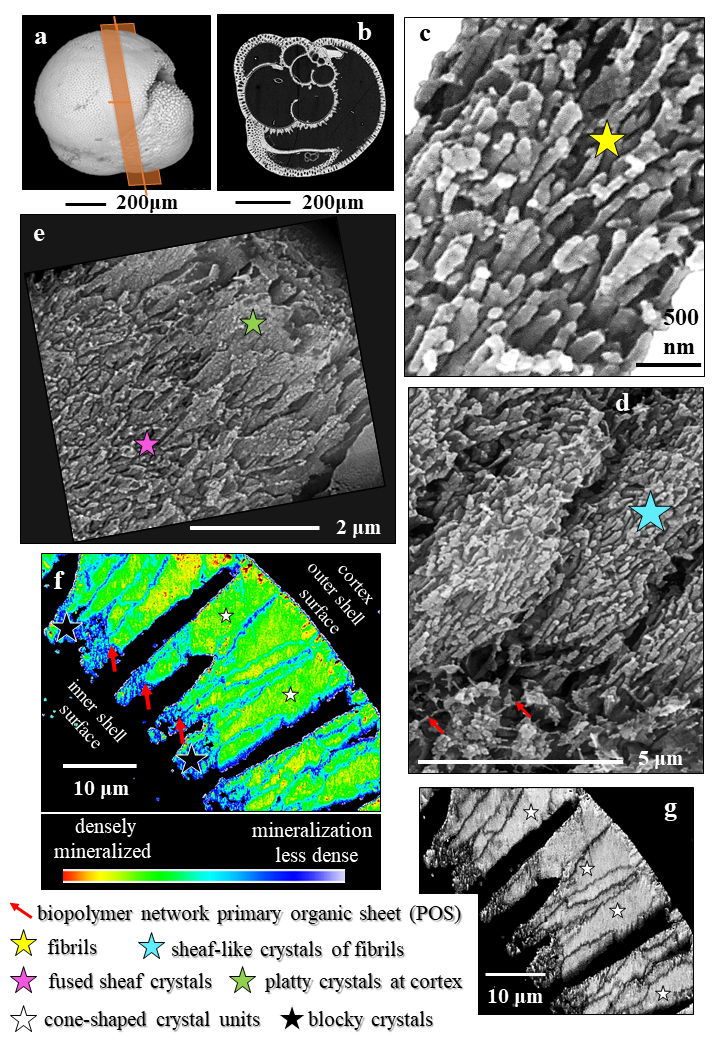
**

**Figure S1.** Figure compiled for illustration of the crystal shape terminology used in this contribution. Visualization of fibril, platy, sheaf-like and cone-shaped crystal morphologies ((c) to (g)). (a): The shell of *P. obliquiloculata* and the mode of sectioning through the shell; BSE contrast. (b): BSE image of the shell cross-section that was used for etching experiments. ((c, (d), (e)): etched shell cross-section surfaces. (f) and (g): EBSD band contrast measurement images given in grey (g) and in color (f), depicting the sites of the POS as well as the sites of small blocky crystals at inner shell surfaces and the seam of blade-shaped crystals forming the cortex at outer shell surface. (g): well visible are the cone-shaped units that comprise the shell wall from the POS up to outer shell surface.


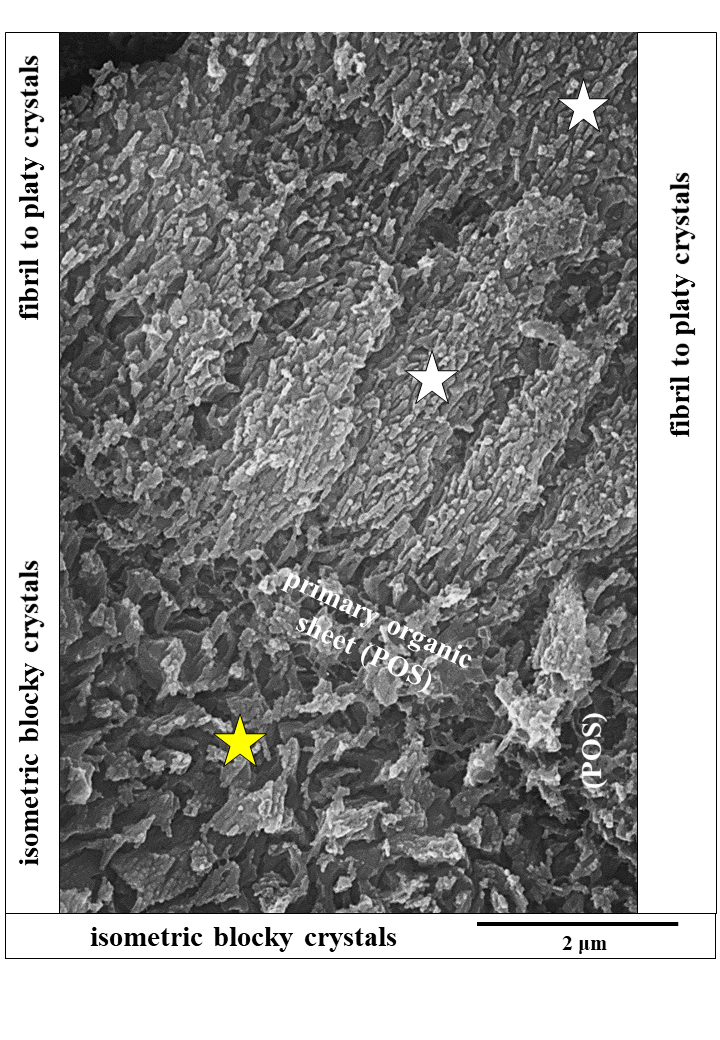


**Figure S2.** BSE image of an etched surface of a sagittal cross-section through the shell wall of *P. obliquiloculata* depicting the two types of crystal morphologies that are present at the two sides of the POS. Yellow star: blocky crystals on the one side of the POS, at inner shell portions. White star: fibril-shaped to fibrous crystals on the other side of the POS, at outer shell sections.


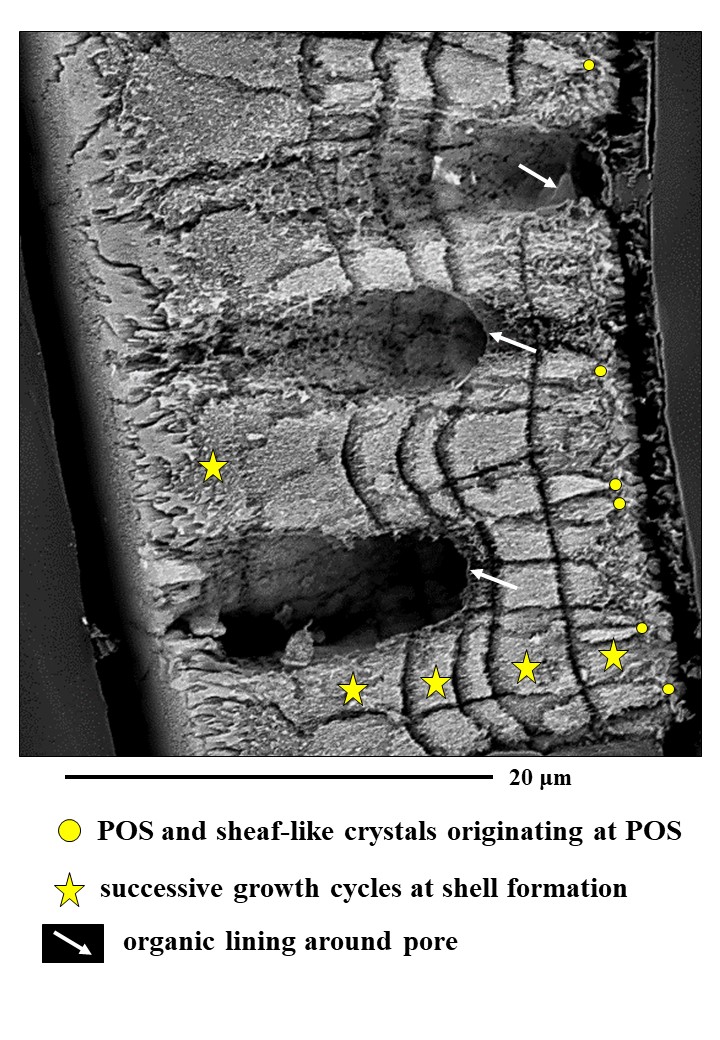


**Figure S3.** BSE image of an etched cross-section through the shell wall of *Pulleniatina obliquiloculata*, sagittal cut. Well observable is the organic lining at the inner surface of the pores (white arrows), the start of growth of the sheaf-like crystals at/in the POS (yellow dots) and traces of organic linings, OOL, (undulating black lines) left behind from the successive chamber formation events (yellow stars). The successively incorporated organic linings, due to being reticulate, do not change the structural and crystallographic characteristics of the crystals. The latter characteristics are steadily transmitted from the POS to the cortex, across the different organic incorporations.


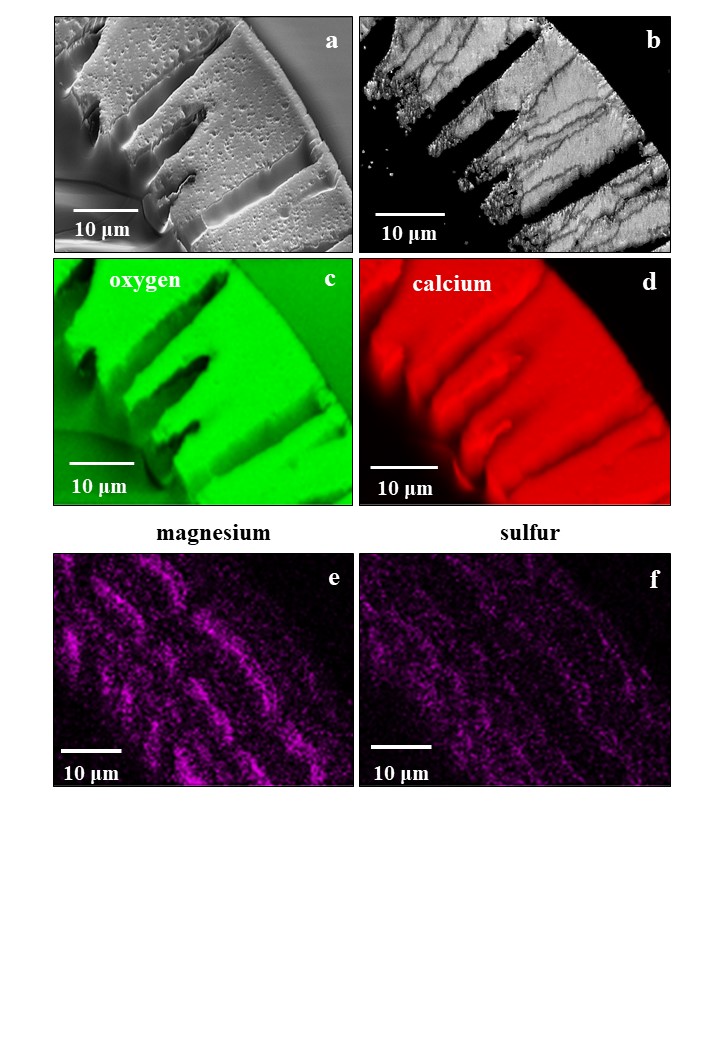


**Figure S4.** Zonation in magnesium (e) and sulfur (f) on a cross-section through *Pulleniatina obliquiloculata* shells. Sulfur is indicative of organic substance. An enrichment of magnesium is correlated to an enrichment in sulfur. (a): SE image; (b): EBSD band contrast measurement in grey; ((c) to (f)): oxygen, calcium, magnesium, sulfur distribution maps.


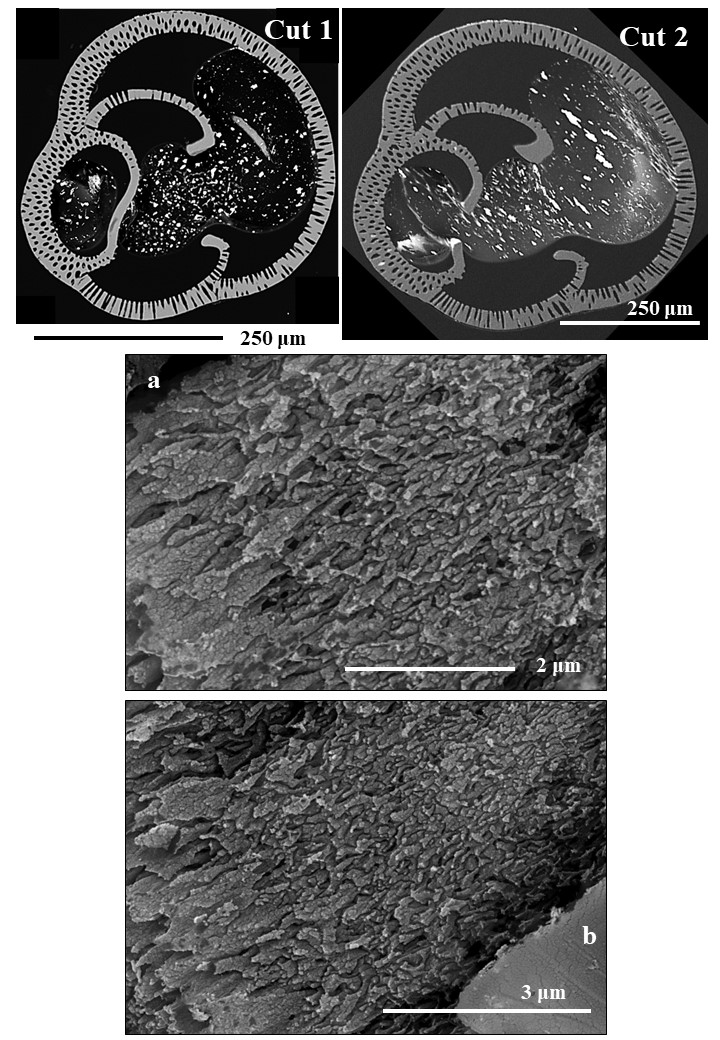


**Figure S5.** Sagittal cross-section through the shell of *P. obliquiloculata*. The shell was sectioned at two different depths. The structural and crystal morphological characteristics that we observe are not just surficial features of the shells, but occur throughout the shells, e.g. at different depths. Cut 1: cut made at upper shell sections; cut 2: cut made below cut 1. (a) and (b): BSE image of the etched surface of cut 1 (a) and that of cut 2 (b).


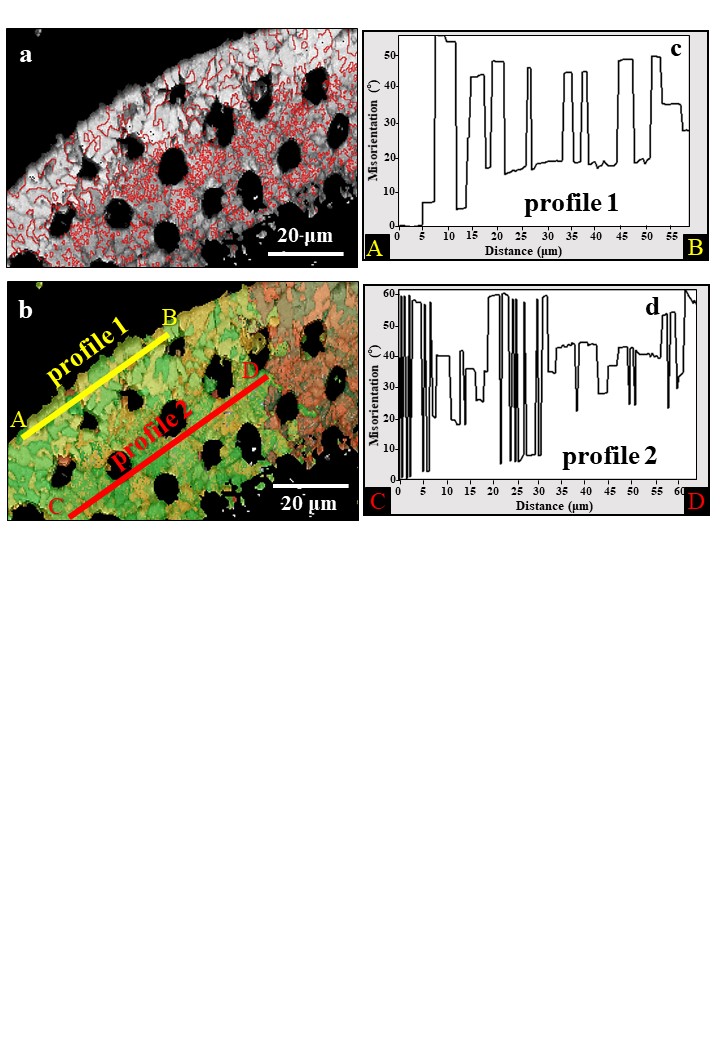


**Figure S6.** Tangentially oriented cuts through the shell of *Pulleniatina obliquiloculata* visualizing: (a): the distribution of 60° grain boundaries (red lines), (b): the jagged morphology and strong interlinkeage of cone-shaped units and (c, d): the degree of misorientation between adjacent cone-shaped entities. As the misorientation versus distance diagrams along profiles A to B (c) and C to D (d) show, neighboring cone-shaped units are misoriented to each other, by variable degrees, and are not related to each other through a twin relationship (the systematic 60° misorientation). (a): EBSD band contrast measurement, given in grey, superimposed with 60° misorientation boundaries, given in red. (b): EBSD scan visualizing in color crystal orientation; the color-code is shown in Fig. 8a. (b): Yellow and red lines indicate the trace of misorientation profiles 1 and 2. Corresponding misorientation versus distance diagrams are presented in Figs. S6c and S6d.


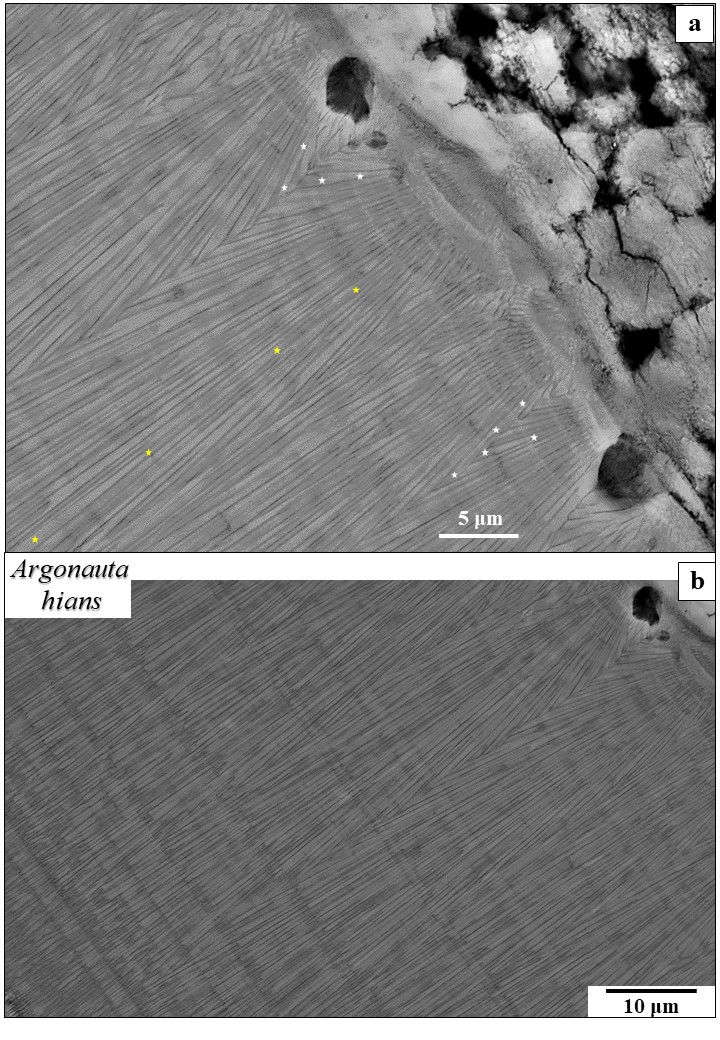


**Figure S7.** BSE image of a cross-section through the shell of the cephalopod mollusk *Argonauta hians* visualizing calcite crystal growth through a competitive growth process. Due to high regularity in crystal morphology, implications of mineral growth through a competitive growth process are exceptionally well visible for *Argonauta* sp. calcite. Crystal nucleation starts within a network of biopolymer fibrils, the latter becomes occluded within the shell at shell growth. Spherulites form first (upper right corner in (a)) and extend gradually in size. Of the spherulites, the calcite on the two sides of the network of biopolymer fibrils forms through crystal competition. Only those crystals survive from the site of nucleation to outer shell surfaces that have the fastest growth direction (their c-axis) oriented normal to the organic template (yellow stars in (a)), where the first calcite seeds nucleate. Crystals with their c-axis being inclined to the organic template become eliminated as they impinge on neighboring crystals that have also their c-axis inclined to the plane, where crystal nucleation starts (white stars in (a)). The result is that (i) crystals at outermost shell surfaces are aligned in parallel (see left-hand lower corner in (b)) and (ii) calcite c-axes are perpendicular to the shell surface.


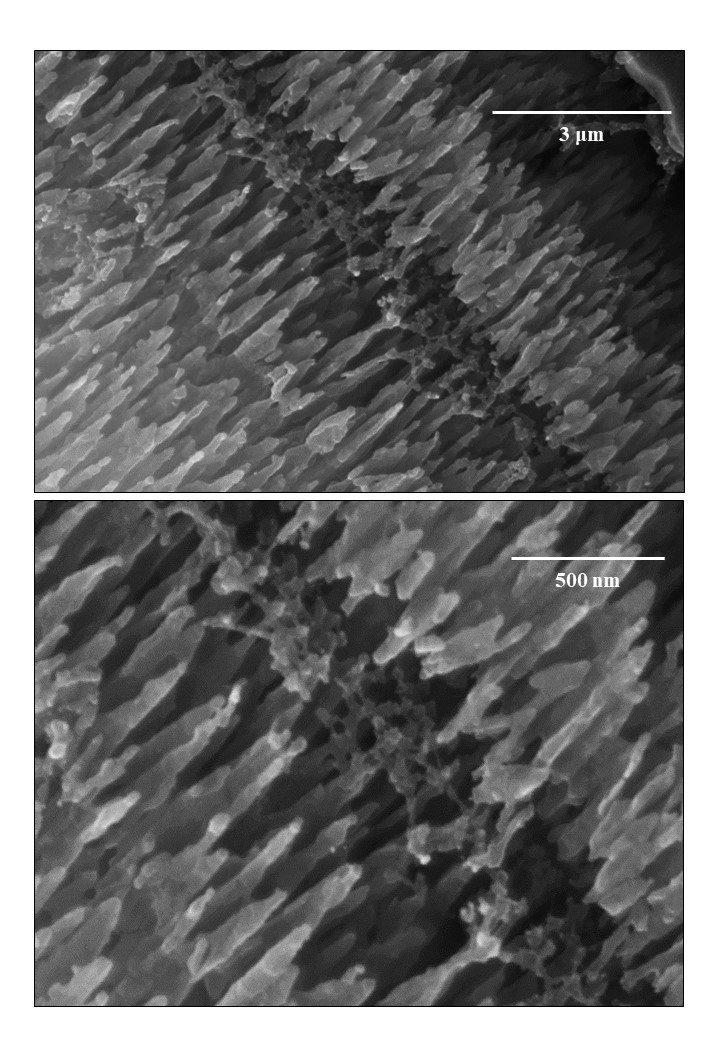


**Figure S8.** BSE images visualizing for the planktonic species *Globigerinita glutinata* the fabric of the outer organic lining (OOL) and demonstrating its reticulate structure. The figure demonstrates the smooth transfer of crystallized mineral across the OOL at resumption/continuation of crystallization.


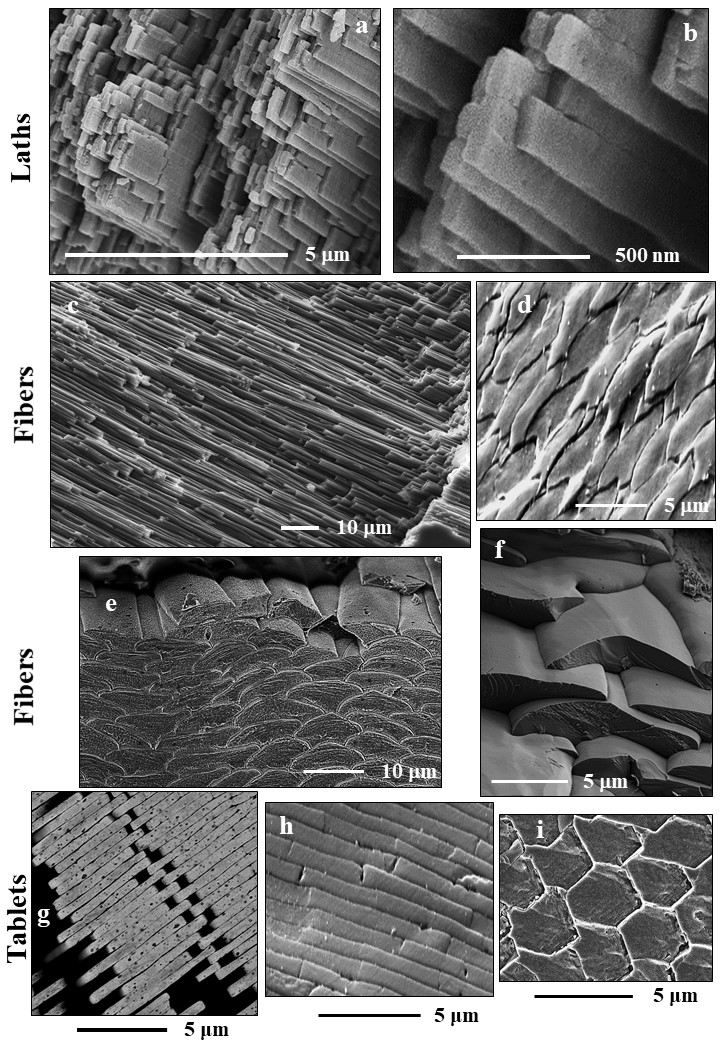


**Figure S9.** BSE images highlighting the regularity of carbonate crystal morphologies that comprise bivalve, gastropod and brachiopod shells. (a, b): assembly of aragonite laths to crossed-lamellar and complex-crossed-lamellar arrangements in *Glycymeris glycymeris* shells. (c, d): assembly of calcite fibers in the calcitic layer of *Mytilus edulis* shells. (e, f): assembly of calcite fibers in the fibrous shell layer of the brachiopods *Laqueus rubellus* (e) and *Notosaria nigricans* (f). (g): columnar arrangement of aragonite tablets in the nacreous shell layer of the gastropod *Haliotis ovina*. (h, i): sheeted assembly of aragonite tablets in the nacreous shell layer of the bivalve *Mytilus edulis*.


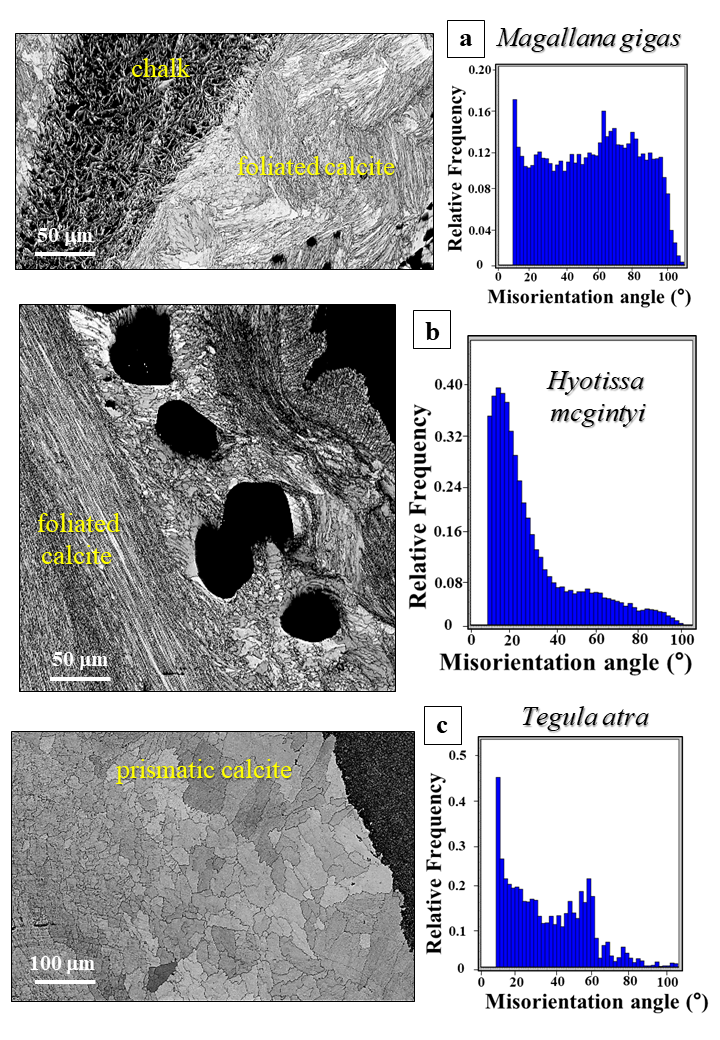


**Figure S10.** EBSD band contrast measurement images (in grey) visualizing calcite crystal assemblies formed of crystals with irregular morphologies. (a) the chalk section in the shell of the oyster *Magallana gigas*, (b) calcite around voids and cavities in the shell of the oyster *Hyotissa mcgintyi*, (c) assemblies of prisms in the shell of the gastropod *Tegula atra*. The corresponding misorientation angle distributions show a wide range of misorientations between the crystals, unlike to what is detected for *P. obliquiloculata* shell calcite, e.g. the relative frequency-misorientation angle diagrams shown in Fig. 7, where we observe, for the twinned calcite, a very prominent peak at 60° misorientation.
